# Effect of conservative treatment on greater trochanteric pain syndrome: a systematic review and network meta-analysis of randomized controlled trials

**DOI:** 10.1186/s13018-025-05477-w

**Published:** 2025-01-31

**Authors:** Si-Qi Wang, Ning-Yi Guo, Wei Liu, Hong-jie Huang, Bing-bing Xu, Jian-quan Wang

**Affiliations:** 1 Department of Sports Medicine and Execise Rehabilitation, Beijing Sport University, Beijing, China; 2 Department of Sports Medicine, Peking University Third Hospital, Institute of Sports Medicine of Peking University, Beijing, China; 3 Beijing Key Laboratory of Sports Injuries, Beijing, China; 4 Engineering Research Center of Sports Trauma Treatment Technology and Devices, Ministry of Education, Beijing, China

**Keywords:** Greater trochanteric pain syndrome (GTPS), Conservative treatment, Exercise, Physical modalities, Injection

## Abstract

**Objective:**

Numerous studies have investigated conservative treatments for GTPS. However, there remains a lack of comparative analyses among treatment modalities, making it challenging to formulate the optimal conservative treatment strategy. This study aimed to compare conservative treatments for greater trochanteric pain syndrome (GTPS) in terms of their effectiveness in relieving pain and improving functional outcomes.

**Methods:**

In accordance with the Preferred Reporting Items for Systematic Reviews and Meta-Analyses guidelines, the PubMed, Embase, Cochrane Library and ClinicalTrials.gov along with other databases were searched to identify relevant studies. The quality of the selected studies was evaluated using risk of bias assessments, and the data were extracted. Network meta-analysis was performed using random effects models to evaluate the effects of treatment modalities on pain reduction and functional improvement. The evidence of the included studies was evaluated using the Grading of Recommendations, Assessment, Development, and Evaluations framework.

**Results:**

Nineteen RCTs involving 1701 participants were included. The risk of bias was assessed using Cochrane ROB2. The results of network meta-analysis revealed that exercise therapy yielded the most significant improvement on reducing pain scores measured using the NRS and enhancing functional outcomes measured using the VISA-G. Additionally, injection therapy showed significant advantages in improving functional outcomes measured by using the Harris Hip Scale.

**Discussion:**

This study confirmed the significant efficacy of exercise programs on pain reduction and functional improvement in GTPS patients. Additionally, we identified the positive effects of injection therapy and physical modalities on managing pain and enhancing functional outcomes among GTPS patients. Owing to the limited number of included studies, comparing the effects of different conservative treatments remains challenging. Future studies should expand the quantity of RCTs to identify the optimal conservative treatment strategy for GTPS.

**Conclusion:**

This study reaffirmed the superiority of exercise therapy in the treatment of GTPS and revealed the effectiveness of injection and physical modalities. As the number of studies increase, we anticipate more reliable conclusions to further optimize conservative treatment strategies for GTPS. Ultimately, our study can help clinicians determine the optimal conservative treatment for GTPS and may spur future research on conservative treatments for tendinopathy.

**Supplementary Information:**

The online version contains supplementary material available at 10.1186/s13018-025-05477-w.

## Introduction

Greater trochanteric pain syndrome (GTPS), also known as greater trochanteric bursitis or “hip sleeve” injury, is a common musculoskeletal disorder that affects the lateral hip region and has been reported to have a prevalence ranging from 10 to 25% in developed countries [1, 2]. In addition, approximately two-thirds of people with GTPS subsequently develop lower back pain or hip osteoarthritis [3]. GTPS is characterized by chronic pain and tenderness around the greater trochanter and is often aggravated when standing, walking, or lying down on the affected side. Middle-aged and older people, especially women, are more prone to developing GTPS, and this disorder can greatly reduce quality of life and hinder daily functioning [4].

The aetiology of GTPS is multifactorial. The disorder primarily results from chronic microtrauma and subsequent aseptic inflammation due to prolonged repetitive stretching of the soft tissues adjacent to the greater trochanter [5]. For example [6], excessive use or repetitive strain on the gluteus minimus and gluteus medius muscles, which are crucial for hip abduction and stability, can lead to tendinopathy or tears that contribute to GTPS. Additionally, friction between the muscles or tendons crossing or attaching to the greater trochanter may cause inflammation in the surrounding bursae, further contributing to the development of GTPS. Furthermore, conditions such as hip impingement or osteoarthritis can exert abnormal pressure on the tissues around the greater trochanter, thereby precipitating GTPS.

Currently, conservative treatments for GTPS include physical modalities, exercise therapy, injection therapy, and lifestyle modifications. More specifically, the treatments may include shock wave therapy, isolated eccentric training, high-intensity slow resistance training, corticosteroid injections, platelet-rich plasma injections, and dry needling therapy [7]. In recent years, several meta-analyses on conservative treatment approaches for GTPS have been published [8–10]. However, owing to limitations such as an insufficient number of relevant studies, variations in the inclusion criteria across studies, and the lack of comprehensive evaluations from multiple dimensions such as pain and functionality, the relative efficacy of these conservative treatments remains unclear. Therefore, a consensus on the optimal treatment approach has not yet been established.

The purpose of this comprehensive network meta-analysis (NMA) was to systematically review and synthesize evidence from randomized controlled trials (RCTs) to compare the therapeutic effects of various conservative treatment modalities for GTPS. Our specific aims were threefold: first, to compare the efficacy of different conservative treatments in terms of alleviating pain and improving functional outcomes among patients with GTPS; second, to rank these treatment options based on their effectiveness in order to identify the optimal conservative approach; and third, to thoroughly investigate potential sources of heterogeneity. Ultimately, we aimed to reveal information that would help identify the optimal conservative treatment approach for GTPS, as well as serve as a theoretical foundation for future research on various conservative treatment modalities for GTPS. The results of our study may also be helpful for researchers studying conservative treatments for other types of tendinopathy.

## Methods

### Registration

This systematic review and NMA were conducted in accordance with the Preferred Reporting Items for Systematic Reviews and Meta-Analyses (PRISMA) statement guidelines [11]. The study protocol was registered prospectively in the International Prospective Register of Systematic Reviews ID:CRD42024541484.

### Literature search strategy

The PubMed, Embase, Cochrane Library, Web of Science, and clinical trials.gov were searched to identify published and registered studies. The U.S. Clinical Trial Registry was searched in May 2024 to identify registered but unpublished studies. For the former search, Medical Subject Heading (MeSH) terms and Entry terms were utilized. Detailed information regarding the search strategy implemented for each database is provided in Appendix S2. The search strategies were based on keywords that were identified using the PICO framework: (P) Population—individuals with greater trochanteric pain syndrome (GTPS); (I) Intervention—conservative treatment methods, including injection therapy, exercise therapy, physiotherapy; (C) Comparator—control groups or sham treatments; (O) Outcome measures—indicators of pain or functional status. Additionally, the reference lists of the selected articles and reviews were manually searched to identify any relevant studies that may have been overlooked during the electronic searches. Relevant English-language studies published before May 2024 were included(Since the databases we searched are all in English as the official language, and English literature has the highest publication volume, authority, and wide recognition, in order to ensure the quality of the included research, we decided to include English literature only).

### Eligibility criteria

The inclusion criteria for studies were as follows: (1) RCTs; (2) included patients with GTPS, gluteal tendinopathy or lateral hip pain regardless of age; the patients were clinically diagnosed by a physiotherapist or medical doctor, and the studies reported follow-up data for at least one outcome measure; (3) included an experimental group receiving conservative treatment such as injection therapy, exercise therapy or physical modalities; (4) compared the intervention group with a blank control, sham treatment or other conservative treatment group; and (5) assessed the degree of pain or functional status before and after the intervention, with no restrictions on the measurement method.

The exclusion criteria were as follows: (1) duplicate publications, literature review papers, letters to the editor, abstracts published in conference proceedings, studiers reporting acute effects of a single intervention session, and animal model studies; (2) examined a combination of different conservative treatments (e.g., injection combined with an exercise intervention, but there was no injection intervention control group or exercise group); or (3) compared different adjunctive manoeuvres to the same treatment, e.g., comparing ultrasound-guided steroid injections to steroid injections.

Two researchers independently screened the retrieved articles on the basis of the inclusion and exclusion criteria. After independently reviewing the titles and abstracts of the included articles, the two authors conducted a full-text review of each article that met the criteria. Multiple publications from the same trial were collated, and the first or most complete report was included. If studies reported data for men and women separately, the data were collated.

### Data extraction

Two researchers independently extracted data from the included studies. Disagreements were resolved by consensus or by consulting a third author. The following data were extracted: first author, year of publication, subject characteristics (number of experimental and control groups, sex, age, degree of pain, index of dysfunction, etc.), intervention information (type of intervention, duration, frequency, cycle, supervised or unsupervised), and the measurement method and unit of reported results. If any data were missing, the authors of the included studies were contacted by email.

After data extraction, we classified the interventions included in each study into three categories: physical modalities, injection therapy, and exercise therapy. physical modalities was defined as treatments primarily employing physical modalities such as sound, light, cold, heat, electricity, magnetism, or water. Injection therapy included various treatments administered via injection, such as corticosteroids and platelet-rich plasma (PRP). Exercise therapy encompassed exercise training methods, including eccentric and concentric exercises.

### Risk of bias and certainy of evidence assessments

Two investigators(Wang-SQ and Guo-NY) independently assessed the risk of bias (ROB) of the included studies using the Cochrane Risk of Bias-2 Tool [12], which includes five domains. Each domain in all trials was assigned a study-level score indicating the level of bias risk: low, high, or unclear. Disagreements between reviewers were resolved through discussion. If consensus could not be reached, a final judgment was provided by a third author (Liu-W).

We assessed the certainty of evidence contributing to the network estimates of the primary and secondary outcomes using the Grading of Recommendations Assessment, Development and Evaluation (GRADE) framework [13].

### Data synthesis and statistical analysis

The pre-to-post changes in the experimental and control groups were pooled to estimate the effects. The interventions were, by definition, heterogeneous, and pairwise meta-analytic estimates are also reported in addition to the network estimates to account for heterogeneity among studies by using STATA 15.1 software (StataCorp, College Station, TX, USA), The heterogeneity was analysed via sensitivity analysis. The random effects model (DerSimonian-Laird) was used to obtain pooled estimates. Standardized mean differences (SMDs) with 95% confidence intervals (CIs) were calculated for the numeric rating scale (NRS), which was the main outcome measure(since there are more studies incorporating pain indicators and a broader range of conservative treatments, it serves as the primary outcome indicator for this study), and the Victoria Institute of Sport—Gluteal score (VISA-G) [14] and Harris Hip Score (HHS), which were the secondary outcome measures. The I^2^ statistic and Cochran's Q test were used to quantify heterogeneity. An I^2^ greater than 50% or a p value of 0.10 or less for the Q test was considered to indicate substantial heterogeneity [15]. We evaluated publication bias by inspecting funnel plots and by performing Begg's test.

STATA 15.1 software (StataCorp, College Station, TX, USA) was used to perform random effects multivariate NMA within a frequentist framework [16] in accordance with the current PRISMA NMA guidelines [11]. Because different tools and units were used to measure pain and dysfunction in the included studies, the SMD was used to represent the effect size for different outcome measures.

The relationships among the three major types of conservative interventions are presented in a network diagram, where the lines connecting nodes represent direct head‒to‒head comparisons between interventions and where the size of each node and the thickness of each line connecting the nodes are proportional to the number of studies. A network contribution graph was constructed to calculate the contribution of each direct comparison.

Transitivity assumptions were assessed by evaluating the inclusion criteria for individual studies, determining whether all participants in the network could be randomly subjected to any intervention, and using consistency models [17]. Transitivity is a key assumption of NMA and refers to the belief that indirect comparisons are valid estimates of unobserved direct comparisons [16] and that all studies have homogenously distributed effect modifiers [18]. Inconsistency factors (IFs) with 95% CIs were calculated to evaluate the consistency of each closed loop, and consistency was indicated by the lower limit of the 95% CI being equal to 0 [19]. The inconsistency model was used to test inconsistency. The consistency model was used when the inconsistency was nonsignificant (*p* > 0.05) [20]. Node-splitting analysis was used to check for local inconsistency, and the results were reliable (*p* > 0.05).

The surface under the cumulative ranking curve (SUCRA) was used to rank and compare the effects of different types of conservative interventions [21]. SUCRA values range from 0 to 100, where 100 indicates the best treatment with no uncertainty and 0 indicates the worst treatment with no uncertainty [22]. Thus, higher SUCRA values indicate a better conservative intervention. To check for NMA publication bias caused by small-scale studies, we generated a network funnel plot and performed a visual analysis of the symmetry.

## Results

### Included studies

**Fig. 1:**
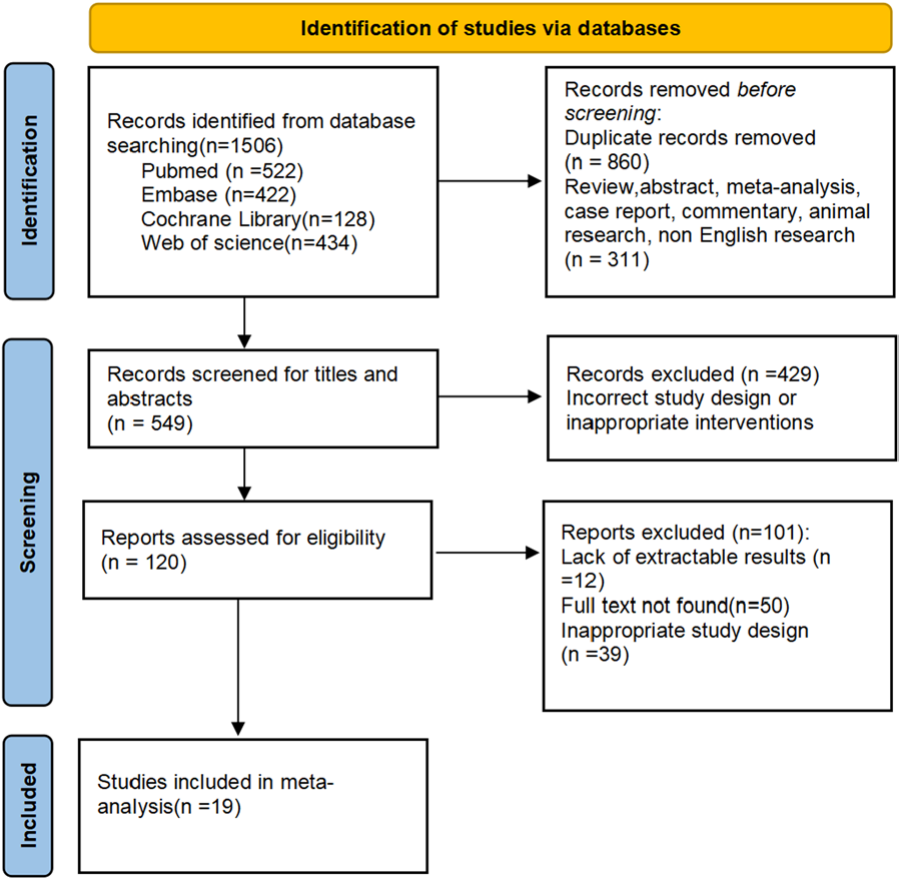
PRISMA flow diagram of the study selection process

The literature search initially yielded 1506 articles. After removing duplicates, 860 articles remained, and these articles were screened based on the inclusion and exclusion criteria. We excluded 311 articles of ineligible types such as reviews and commentaries, as well as animal studies. After reviewing the titles and abstracts, we excluded an additional 429 articles due to flawed study designs or inappropriate interventions. Then, we reviewed the full texts of 120 studies and excluded 101 articles either due to unavailable data or flawed experimental designs. Ultimately, 19 RCTs examining conservative treatment methods for GTPS were included [23–41], comprising a total of 1701 participants. The included studies examined a total of 8 conservative (nonoperative) treatments, which we categorized into three main groups(We only categorize the 8 treatment methods included in the literature, without defining concepts such as physical modalities and injection therapy): physical modalities (dry needling, extracorporeal shockwave therapy, and ultrasonic therapy), exercise therapy, and injection therapy (corticosteroid injections, platelet-rich plasma injections, hyaluronic acid, and bone marrow aspirate concentrate). The flowchart depicting the process of selecting the included studies is shown in Fig. 1.

### Characteristics of the included studies

Supplemental Table 2 presents the characteristics of all included studies. All 19 of the included studies were published between 2009 and 2023. The total sample size across the studies was 1701 participants, of whom 82.5% were female. The following interventions were examined in the included studies: corticosteroid injection (CSI, n = 3), extracorporeal shockwave therapy (ESWT, n = 7), exercise (EX, n = 5), platelet-rich plasma injection (PRP, n = 4), dry needling (DN, n = 1), hyaluronic acid (HA, n = 1), and bone marrow aspirate concentrate (BMAC, n = 1). The control interventions included control, sham exercise, or placebo (CON, n = 7), CSI (n = 9), EX (n = 2), and ultrasound therapy (UST, n = 1). The agreement between the two assessors was 85.1% and 87.0% for the study selection and data extraction processes, respectively.

### Quality assessment of the included studies

**Fig. 2:**
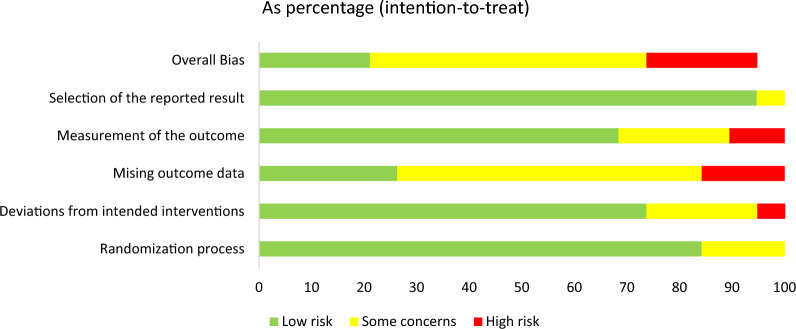
Risk of bias assessment for included study

The overall ROB for the included studies is shown in Fig. 2. Among all 19 studies, 16 (84.2%) had a low risk with respect to selection of the reported result. Thirteen studies (68.4%) appropriately described the measurement of outcome, four studies had some concerns and one had high risk. Additionally, 3 studies had high risk with respect to missing outcome data, and 11 had some concerns. The majority of the included RCTs had a low risk with respect to deviations from intended interventions(73.7%, n = 14). In summary, 4 articles were judged to be of low risk, 10 were of some concerns, and 4 were of high risk. The Risk of bias assessment for every included study is shown in Supplement Fig. 1.

### Evaluation of intervention efficacy

#### Primary outcome measure: numeric rating scale (NRS)

**Fig. 3:**
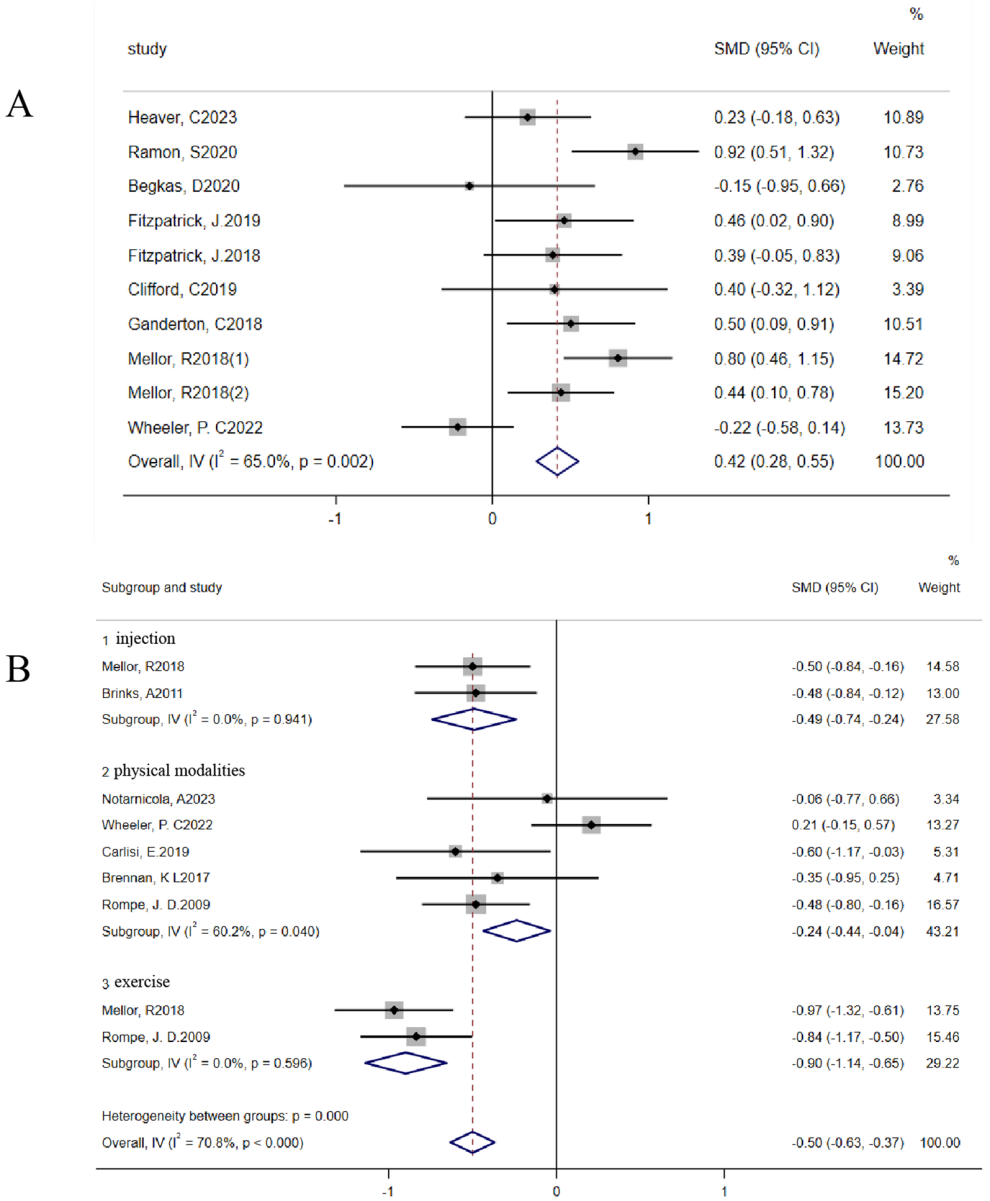
Heterogeneity analysis of pain indicators. **a** Heterogeneity analysis conducted on all studies involving various pain measures. **b** Subgroup analysis of studies reporting NRS pain scores categorized by intervention, with forest plots illustrating the differences in NRS pain scores between the three intervention subgroups and the control group. Subgroup 1: injection therapy (including corticosteroid injections and platelet-rich plasma injections); Subgroup 2: physical modalities (including dry needling and extracorporeal shockwave therapy); Subgroup 3: exercise (exercise therapy)

A total of 11 studies [23–26, 29, 30, 33, 36, 38, 40, 41] reported pain outcomes, and a heterogeneity analysis revealed that there was significant heterogeneity among the studies (Fig. 3a). Subsequent subgroup analyses based on different outcome measures and control groups did not substantially reduce this heterogeneity (Supplemental Fig. 2). Finally, subgroup analyses were performed on different intervention measures based on pain assessment criteria. Owing to the limited number of studies using the VAS, we could not conduct a subgroup analysis of the VAS scores. Therefore, our investigation focused solely on the NRS. The results revealed that there were significant reductions in NRS scores across different intervention measures compared with controls (injection vs. control, SMD − 0.49 [95% CI − 0.74 to − 0.24]; physical modalities versus control, SMD − 0.24 [95% CI − 0.44 to − 0.04]; exercise vs. control, SMD − 0.90 [95% CI − 1.14 to − 0.65]) (Fig. 3b). Particularly in the injection and exercise therapy subgroups, each study demonstrated significant reductions in NRS scores without any heterogeneity. However, heterogeneity was observed within the physical modalities subgroup (I^2^ = 60.2%, *p* for heterogeneity = 0.040).

#### Secondary outcome measure: Harris hip score (HHS)

**Fig. 4:**
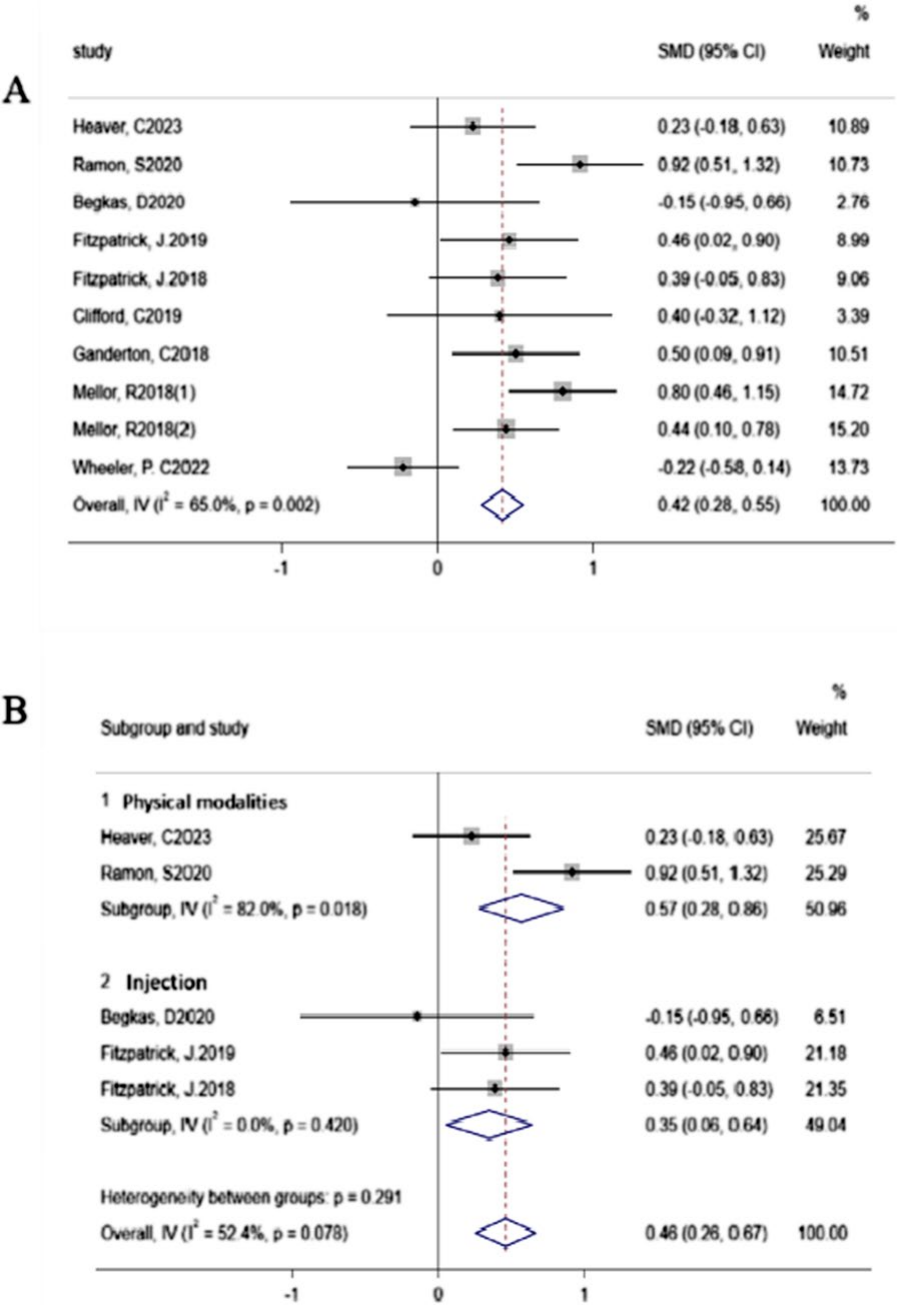
Analysis of heterogeneity in functional indicators. **a** Heterogeneity analysis conducted on all studies involving various functional indicators. **b** Subgroup analysis of heterogeneity in studies involving the HHS categorized by intervention, with forest plots illustrating the differences in HHS scores between the two intervention subgroups and the control group. Subgroup 1: physical modalities (including extracorporeal shockwave therapy); Subgroup 2: injection therapy (including corticosteroid injections, platelet-rich plasma injections)

A total of 9 studies [24, 26, 29–31, 34–37] reported functional indicators, and a heterogeneity analysis revealed that there was significant heterogeneity among these studies (Fig. 4a). Subsequent subgroup analyses based on different indicators and control groups did not substantially reduce this heterogeneity (details in Supplement Fig. 3). Finally, we performed subgroup analyses on different intervention measures based on functional indicators. Owing to the various interventions used in studies that reported the VISA-G—with only one study each for injection therapy and physical modalities—we did not conduct subgroup analysis on the VISA-G indicators. Instead, we focused on HHS scores. The results revealed that both injection therapy and physical modalities led to significant improvements in HHS scores (physical modalities vs. control, SMD 0.57 [95% CI 0.28–0.86]; injection versus control, SMD 0.35 [95% CI 0.06–0.64]) compared with control interventions (Fig. 4b). There was no heterogeneity in the injection therapy subgroup, while there was significant heterogeneity in the physical modalities subgroup (I^2^ = 82.0%, *p* for heterogeneity = 0.018).

### Results of the NMAs

#### Primary outcome measure: numeric rating Scale (NRS)

**Fig. 5:**
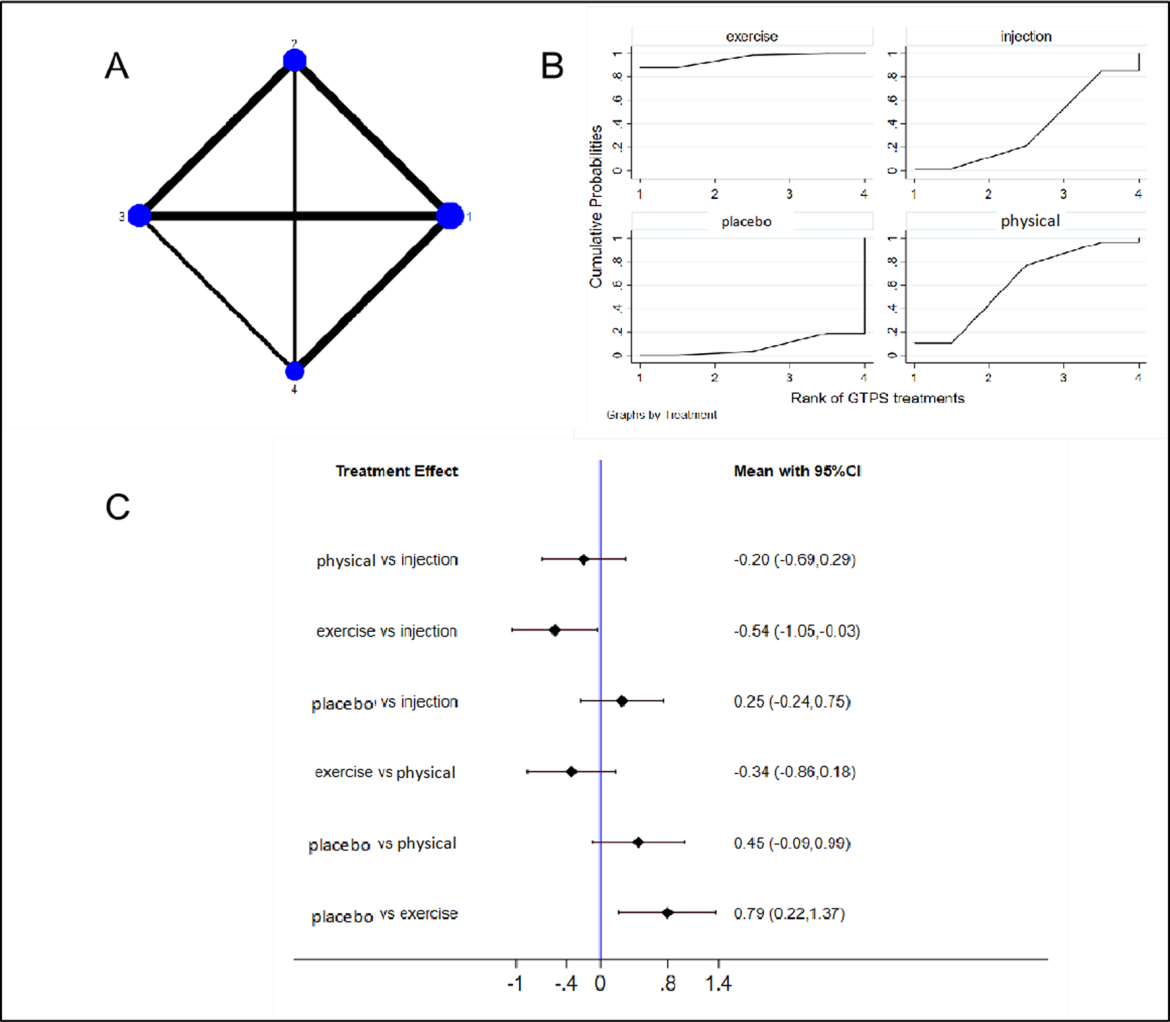
Network diagrams (**a**), cumulative probability graphs (**b**), and forest plots (**c**) for the NRS for pain. 1 Exercise, exercise therapy; 2 Injection, injection therapy (including PRP and CSI); 3 Placebo, sham treatment; and 4 Physical, physical modalities. NRS, Numeric Rating Scale

NMA was conducted to examine 6 studies [25–27, 38, 40, 41] (involving 796 patients) that examined NRS pain scores. These patients received injection therapy (n = 4), physical modalities (n = 4), exercise therapy (n = 3), and placebo or sham treatment (n = 3). Figure 5 presents the NMA plot for NRS scores (Fig. 5a), cumulative probability graphs (Fig. 5b), and forest plots comparing interventions (Fig. 5c). Table 1 displays the SUCRA rankings based on cumulative probability diagrams. The cumulative probability graphs and SUCRA rankings indicate that exercise was the most effective intervention for improving NRS pain indicators among GTPS patients (SUCRA 95.9%; placebo vs. exercise, MD 0.79 [95% CI 0.22–1.37]), followed by physical modalities (SUCRA 61.2%; placebo vs. physical, MD 0.45 [95% CI − 0.09 to 0.99]).

**Table 1:** NRS efficacy ranking table (treatment-relative ranking)

Treatment	SUCRA (%)	PrBest	MeanRank
Injection	35.8	1.0	2.9
Physical modalities	61.2	9.7	2.2
Exercise	95.9	89.0	1.1
Placebo	7.1	0.3	3.8

#### Secondary outcome measures: Victoria Institute of sport–gluteal score (VISA-G) and harris hip score (HHS)

**Fig. 6:**
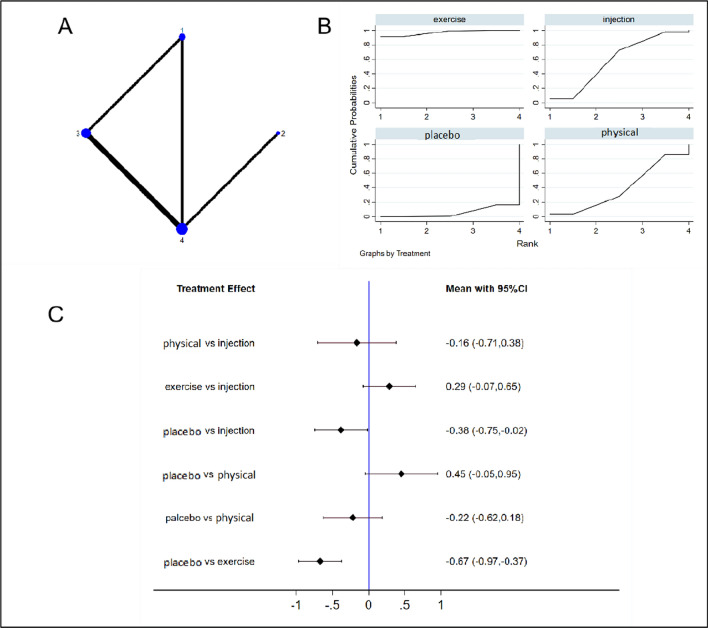
Network diagrams (**a**), cumulative probability graphs (**b**), and forest plots (**c**) for VISA-G. 1 Exercise, exercise therapy; 2 Injection, injection therapy (including corticosteroid injections and platelet-rich plasma injections); 3 and Placebo, sham treatment; 4 and Physical, physical modalities. VISA-G, Victoria Institute of Sport—Gluteal score

NMA was conducted to examine 3 studies [26, 35, 36] (involving 418 patients) that examined VISA-G functional indicators. These patients received injection therapy (n = 1), physical modalities (n = 1), exercise therapy (n = 2), and placebo or sham treatment (n = 3). Figure 6 displays the NMA plot for VISA-G (Fig. 6a), cumulative probability graphs (Fig. 6b), and forest plots comparing interventions (Fig. 6c). Table 2 presents the SUCRA rankings based on the cumulative probability diagrams. The cumulative probability graphs and SUCRA rankings indicated that exercise therapy was the most effective intervention in terms of improving VISA-G functional indicators for GTPS patients (SUCRA 96.9%; placebo vs. exercise, MD − 0.67 [95% CI − 0.97 − 0.37]), followed by injection therapy (SUCRA 58.4%; placebo vs. injection, MD − 0.38 [95% CI − 0.75 − 0.02]).

**Table 2:** VISA-G efficacy ranking table (treatment-relative ranking)

Treatment	SUCRA (%)	PrBest	MeanRank
Injection	58.4	4.9	2.2
physical modalities	39.2	3.7	2.8
Exercise	96.9	91.4	1.1
Placebo	5.5	0.0	3.8

**Fig. 7:**
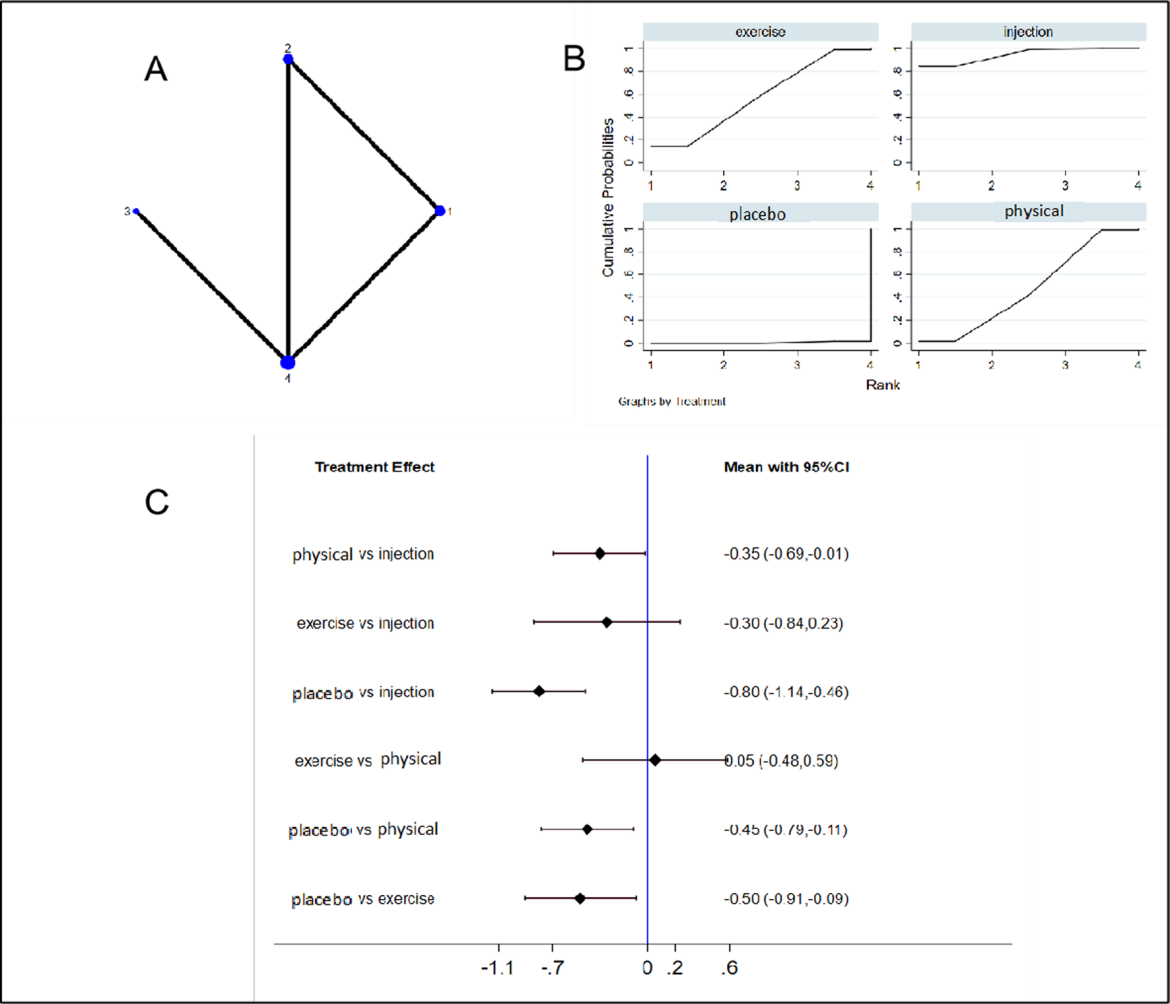
Network diagrams (**a**), cumulative probability graphs (**b**), and forest plots (**c**) for the HHS. 1 Exercise, exercise therapy; 2 Injection, injection therapy (including corticosteroid injections and platelet-rich plasma injections); 3 Placebo, sham treatment; 4 Physical, physical modalities; HHS, Harris Hip Score

NMA was also conducted on 4 studies [24, 26, 35, 36] (involving 370 patients) that reported the HHS. These patients received injection therapy (n = 1), physical modalities (n = 1), exercise therapy (n = 2), and placebo or sham treatment (n = 3). Figure 7 displays the NMA plot for the HHS (Fig. 7a), cumulative probability graphs (Fig. 7b), and forest plots comparing interventions (Fig. 7c). Table 3 presents the SUCRA rankings based on the cumulative probability diagrams. The cumulative probability graphs and SUCRA rankings indicate that injection therapy was the most effective intervention in terms of improving the HHS among GTPS patients (SUCRA 94.5%; placebo vs. injection, MD − 0.80 [95% CI − 1.14 to − 0.46]), followed by exercise therapy (SUCRA 58.4%).

**Table 3:** HHS efficacy ranking table (treatment-relative ranking)

Treatment	SUCRA (%)	PrBest	MeanRank
Injection	94.5	84.3	1.2
physical modalities	47.6	1.5	2.6
Exercise	57.4	14.2	2.3
Placebo	0.5	0.0	4.0

#### Publication bias

**Fig. 8:**
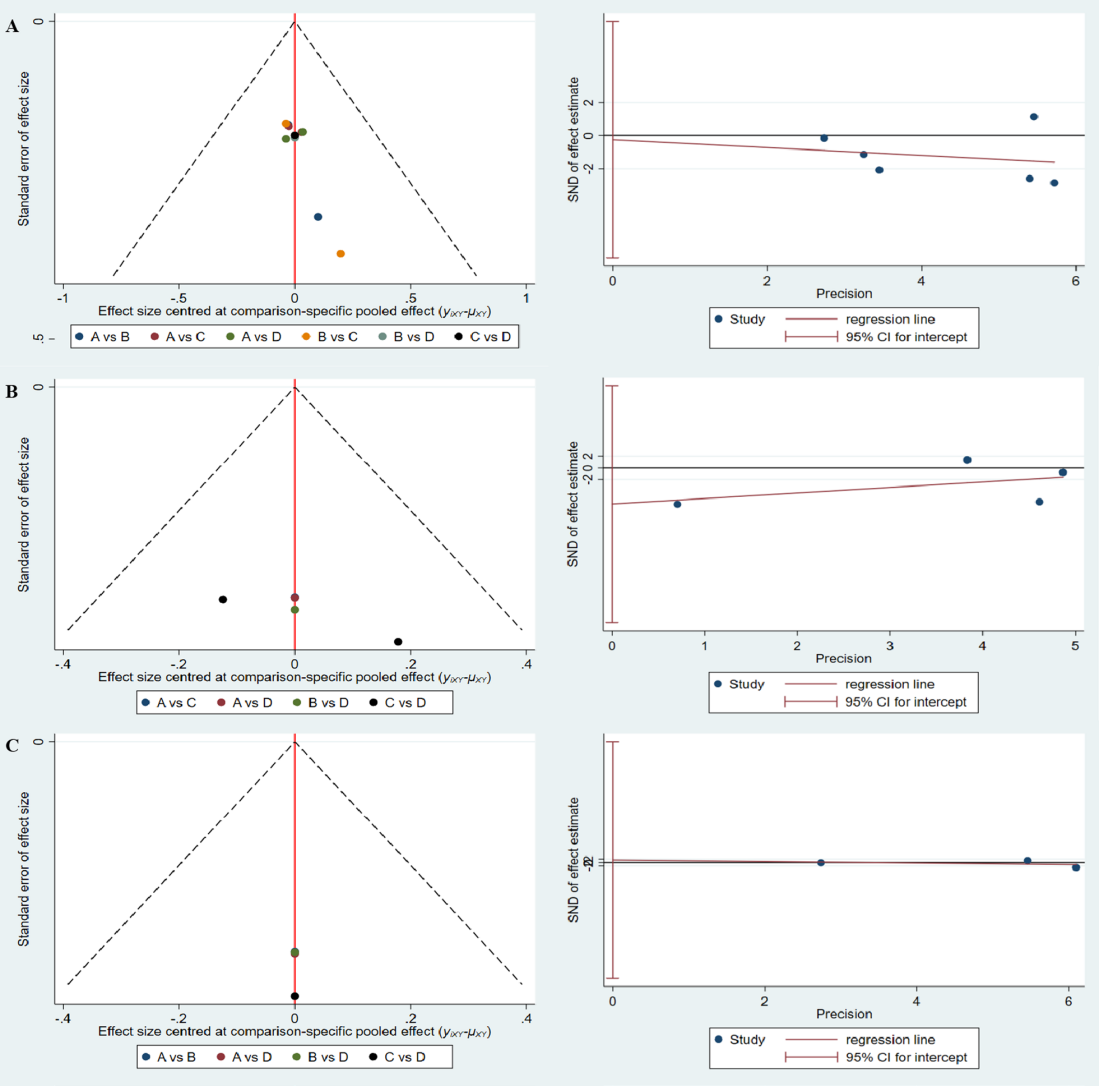
Publication bias funnel plots and Egger's test results for **a** NRS, **b** VISA-G, **c** HHS

We examined the degree of publication bias for the primary and secondary outcome measures in this NMA, including the NRS, VISA-G, and HHS scores. The funnel plots of all outcomes were symmetric, indicating a low possibility of publication bias or a small sample effect in the NMA, and Egger's test showed no publication bias in all subgroups. The results are depicted in Fig. 8.

## Discussion

This study is the first NMA to compare conservative treatment regimens for GTPS. The NMA chart and SUCRA rankings were obtained using combined data from 19 studies. Unlike previous network meta-analyses of GTPS treatment, this study emphasizes the inclusion of RCTs and provides a more comprehensive evaluation of the efficacy of various conservative treatments in terms of both pain and function among patients with GTPS. The results showed that while most interventions were more beneficial than placebo or sham treatments in improving pain and functional outcomes in patients with GTPS, specific effects varied significantly by intervention.

In the heterogeneity analysis, the subgroup analysis based on the type of intervention effectively reduced intra-group heterogeneity after specific pain and function scales were distinguished. However, in the subgroup analysis of NRS pain outcomes, the physical modalities subgroup showed a relatively higher level of heterogeneity; this analysis included two studies [38, 41] that used injection therapy, one study that used exercise therapy [25], and two studies [26, 33] that used physical modalities as control measures, which may have contributed to the increased heterogeneity in that subgroup. The results of the NMA showed that exercise therapy was the most effective intervention in improving the NRS pain score and VISA-G functional indicators, while injection therapy was the most effective intervention in improving the HHS score. This NMA, based on published studies that were available for inclusion, reveals that three main interventions for GTPS patients can improve pain or functional measures, with exercise therapy being the most effective treatment.

Previous studies have shown that exercise therapy exerts therapeutic effects on various tendinopathies, including GTPS [43–46]. Exercise therapy is currently considered the most effective conservative treatment for tendinopathies [47]. Patients with GTPS typically exhibit significant weakness of the hip abductor muscles and gluteal atrophy, leading to decreased pelvic stability and biomechanical alterations [48]. This decreased stability exacerbates the load on the hip abductor tendon group, causing pain and functional impairment. Research has shown that correcting weaknesses in the hip abductor muscles through exercise therapy can restore their role in stabilizing the pelvis, thereby improving pain during movement in patients [49]. Notably, 82.5% of the patients included in this meta-analysis were female. Female sex is a widely recognized risk factor for GTPS, possibly because of the unique biomechanical characteristics of the female pelvis [50] that lead to increased stress on the gluteus medius and minimus tendons during complex lower limb movements, thereby increasing the risk of lateral hip pain among females. Many researchers believe that strength training and appropriate stretching can correct abnormal pelvic biomechanical structures in female patients, strengthen their muscle groups, and thus improve the functional ability and pain levels of GTPS patients. Mellor R’s high-quality RCTs included in this meta-analysis showed that exercise training is superior to traditional CSIs in terms of improving pain and function in patients over both short-term (8 weeks) and long-term (52 weeks) follow-ups [28, 37]. Despite the general consensus on the effectiveness of exercise therapy for tendinopathies [51], various types of exercise programs, including isometric contractions, isolated eccentric training, combinations of eccentric and concentric contractions, or heavy slow resistance training (HSR), lack standardized criteria or guidelines for the intensity and duration of training for patients [52–54]. Moreover, owing to inherent characteristics of exercise therapy, such as challenges in achieving strict supervision and ensuring patient compliance, as well as inconsistencies in exercise modalities, facilities, and equipment standards, there remains no consensus on how to develop optimal exercise training programs for patients with GTPS. A recent review article refined the classification of GTPS conditions [55], proposing an extension beyond traditional contractile/noncontractile classifications [56,57] to categorize GTPS into high/low irritability subtypes. Tailored exercise training programs have been proposed for each subtype. We believe this represents a significant advancement in optimizing exercise training programs for GTPS and other tendinopathies. Further personalization of patient differentiation based on individual criteria could lead to customized exercise training plans, thereby potentially improving outcomes for GTPS patients.

Physical modalities methods for tendinopathy encompass various specific treatment modalities, among which ESWT has been extensively studied in patients with GTPS. ESWT delivers high-energy shock waves through the skin to target tissues, thus promoting the expression of various growth factors and increasing local blood flow, thereby improving nonsurgical treatment options for musculoskeletal tendon diseases [55,57]. ESWT has demonstrated therapeutic effects in several studies on various tendon diseases, such as insertional and non-insertional Achilles tendinopathy [58], rotator cuff tendinopathy [59], and lateral elbow tendinopathy [60], but few high-quality RCTs have specifically considered the use of ESWT among GTPS patients. Moreover, the parameters used in the ESWT studies included herein, such as frequency and energy, vary significantly, making it challenging to determine the optimal treatment parameters for GTPS patients. In addition, for other tendinopathies, such as Achilles tendinopathy, lateral elbow tendinopathy, and rotator cuff tendinopathy, numerous high-quality RCTs have shown significant therapeutic effects of physical modalities modalities, such as low-level laser therapy [61–63] and ultrasound therapy [64]. However, research on these physical modalities modalities in GTPS is notably scarce. We speculate that this may be due to the following reasons. (1) The superficial nature and lower degree of muscle coverage of joints, such as the ankle and elbow joints, facilitate the application and effectiveness of physical modalities modalities. (2) The complex aetiology of GTPS, whereby many patients experience degeneration of multiple tendons around the hip joint rather than isolated tendon injuries in one or two muscle groups, poses significant challenges in designing physical modalities protocols for GTPS patients. Therefore, additional high-quality RCTs should examine the effectiveness of various physical modalities modalities in GTPS patients in order to draw more accurate conclusions.

Injection therapy is the traditional treatment for tendon diseases such as GTPS. Multiple studies have shown that CSIs, which are widely used for immediate pain relief, reduce tendon cell proliferation and collagen synthesis capacity. This interference with the local tissue physiological healing process leads to increased collagen necrosis and decreased mechanical properties of the tendons [65,66]. Therefore, CSIs may not fundamentally improve the condition or prognosis of tendinopathy and reduce the need for subsequent surgical intervention. However, this does not diminish the clinical value of CSIs. For patients with GTPS, CSIs can provide significant short-term pain relief, thereby improving function and promoting participation in recovery. An emerging alternative to CSIs is PRP therapy. Basic studies have shown that PRP releases multiple growth factors from platelet alpha-particles, including TGFβ, PDGF, bFGF, and VEGF, all of which play a role in different stages of tendon healing and theoretically promote musculoskeletal tissue regeneration. However, many RCTs on various tendinopathies have not shown a significant difference in effectiveness between PRP and placebo treatment [33]. In addition, several meta-analyses and systematic reviews do not show significantly higher efficacy of PRP in treating tendinopathy [67–69], leading to conclusions that refute PRP as a first-line treatment for these diseases. Despite these findings, the theoretical feasibility of PRP for the treatment of tendinopathy has not yet been fully realized in practical applications. This gap may be due to a lack of consensus on the optimal concentrations and preparation methods for PRP components in current blood formulations. In addition, the lack of specificity of PRP formulations and injection techniques for various types of tendinopathy may affect their potential therapeutic outcomes.

## Conclusion

In summary, findings of our NMA revealed that structured exercise programs significantly improve pain and functional outcomes in GTPS patients. Exercise training is the optimal treatment approach for various tendinopathies, including GTPS. Additionally, we found that injections and physical modalities exerted beneficial effects on pain and functional outcomes among GTPS patients. However, owing to the limited number of RCTS on nonsurgical treatments for GTPS, comparisons of the effectiveness of different conservative therapies remain challenging. We anticipate that ongoing research efforts will yield more specific and credible conclusions regarding the optimal conservative treatments for GTPS as study volumes continue to grow in the future. Ultimately, our study can help clinicians determine the optimal conservative treatment for GTPS and provide insights for future research on conservative treatments for other tendon diseases.

## Limitations

1. There is significant variability among different types of conservative treatment approaches, and various assessment scales are used for evaluating outcomes such as pain and function in tendinopathy patients. Consequently, the inclusion of the 19 studies itself resulted in substantial heterogeneity.
2. Although our subgroup analyses targeting intervention measures largely mitigated heterogeneity within each subgroup, differences in specific intervention methods among studies within subgroups still contributed to some degree of within-group heterogeneity. For instance, due to the inclusion of various treatment modalities such as ultrasound therapy and extracorporeal shock wave therapy in the physical modalities subgroup, there remains a certain degree of heterogeneity within this subgroup. Furthermore, subgroups within the same treatment method encompass a variety of control treatment measures, which, to some extent, also contributes to heterogeneity and bias.
3. Currently, there is a lack of large-scale RCTs, resulting in a limited number of studies included in our NMA for certain conservative treatment approaches in GTPS. Continuous updates of data regarding these treatment approaches are necessary to increase the credibility of the results derived from NMA.
4. Owing to the nature of treatment methods such as exercise training, blinding of patients was not feasible in some studies, which partly affected the analysis outcomes of this paper.
5. This NMA and systematic review included only studies published in English, and we excluded some non-English literature during the screening process, which to some extent led to information gaps and contributed to heterogeneity.
6. This NMA and systematic review excluded studies that simultaneously applied multiple conservative treatments to the same group of patients in order to ascertain the therapeutic efficacy of individual conservative treatments. In reality, the concurrent use of multiple therapies is quite prevalent in clinical practice, potentially leading to biases.

## Supplementary Information

Supplementary material 1.

## Data Availability

No datasets were generated or analysed during the current study.
