# Supplementary material for: Effect of conservative treatment on greater trochanteric pain syndrome: a systematic review and network meta-analysis of randomized controlled trials
Source: J Orthop Surg Res. 2025 Jan 31;20:126. doi: 10.1186/s13018-025-05477-w (PMC11783921; doi:10.1186/s13018-025-05477-w)
Supplement: Supplementary file 1 — Supplementary material 1. [file 13018_2025_5477_MOESM1_ESM.docx]

### **Supplementary material**

**Supplemental Table 1.** PRISMA NMA checklist of items to include when reporting a systematic review involving a network meta-analysis.

| **Section/Topic** | **Item #** | **Checklist Item** | **Reported on Page #** |
| --- | --- | --- | --- |
| **TITLE** |  |  |  |
| Title | 1 | Effect of conservative treatment on patients with greater trochanteric pain syndrome, A Systematic Review and Network Meta-Analysis of Randomized Controlled Trials | 1 |
| **ABSTRACT**  Structured summary  **INTRODUCTION** | 2 | Provide a structured summary including, as applicable: **Background:** main objectives  **Methods:** data sources; study eligibility criteria, participants, and interventions; study appraisal; and *synthesis methods, such as*  *network meta-analysis.*  **Results:** number of studies and participants identified; summary estimates with corresponding confidence/credible intervals;  *treatment rankings may also be discussed. Authors may choose to summarize pairwise comparisons against a chosen treatment*  *included in their analyses for brevity.*  **Discussion/Conclusions:** limitations; conclusions and implications of findings.  **Other:** primary source of funding; systematic review registration number with registry name. | 1 |
| Rationale | 3 | Describe the rationale for the review in the context of what is  already known*, including mention of why a network meta-analysis has been conducted.* | 1-2 |
| Objectives  **METHODS** | 4 | Provide an explicit statement of questions being addressed, with  reference to participants, interventions, comparisons, outcomes, and study design (PICOS). | 2 |
| Protocol and registration | 5 | Indicate whether a review protocol exists and if and where it can be accessed (e.g., Web address); and, if available, provide registration information, including registration number. | 2 |
| Eligibility criteria | 6 | Specify study characteristics (e.g., PICOS, length of follow-up) and report characteristics (e.g., years considered, language, publication status) used as criteria for eligibility, giving rationale. *Clearly*  *describe eligible treatments included in the treatment network, and note whether any have been clustered or merged into the same node (with justification).* | 2 |
| Information sources | 7 | Describe all information sources (e.g., databases with dates of  coverage, contact with study authors to identify additional studies) in the search and date last searched. | 2-3 |
| Search | 8 | Present full electronic search strategy for at least one database, including any limits used, such that it could be repeated. | 3 |
| Study selection | 9 | State the process for selecting studies (i.e., screening, eligibility, included in systematic review, and, if applicable, included in the meta-analysis). | 3 |
| Data collection process | 10 | Describe method of data extraction from reports (e.g., piloted forms, independently, in duplicate) and any processes for obtaining and  confirming data from investigators. | 3 |
| Data items | 11 | List and define all variables for which data were sought (e.g.,  PICOS, funding sources) and any assumptions and simplifications made. | 3 |
| Geometry of the network | S1 | Describe methods used to explore the geometry of the treatment  network under study and potential biases related to it. This should  include how the evidence base has been graphically summarized for  presentation, and what characteristics were compiled and used to  describe the evidence base to readers. | 3 |
| Risk of bias  within individual  studies | 12 | Describe methods used for assessing risk of bias of individual  studies (including specification of whether this was done at the  study or outcome level), and how this information is to be used in  any data synthesis. | 3 |
| Summary  measures | 13 | State the principal summary measures (e.g., risk ratio, difference in  means). Also describe the use of additional summary measures  assessed, such as treatment rankings and surface under the  cumulative ranking curve (SUCRA) values, as well as modified  approaches used to present summary findings from meta-analyses. | 3 |
| Planned methods  of analysis | 14 | Describe the methods of handling data and combining results of  studies for each network meta-analysis. This should include, but not  be limited to:  • Handling of multi-arm trials;  • Selection of variance structure;  • Selection of prior distributions in Bayesian analyses; and  • Assessment of model fit. | 3-4 |
| Assessment of  Inconsistency | S2 | Describe the statistical methods used to evaluate the agreement of  direct and indirect evidence in the treatment network(s) studied.  Describe efforts taken to address its presence when found. | 3-4 |
| Risk of bias  across studies | 15 | Specify any assessment of risk of bias that may affect the  cumulative evidence (e.g., publication bias, selective reporting  within studies). | 3-4 |
| Additional  analyses | 16 | Describe methods of additional analyses if done, indicating which  were pre-specified. This may include, but not be limited to, the  following:  • Sensitivity or subgroup analyses;  • Meta-regression analyses;  • *Alternative formulations of the treatment network; and*  • *Use of alternative prior distributions for Bayesian analyses*  *(if applicable).* | 4 |
| **RESULTS** | | | |
| Study selection | 17 | Give numbers of studies screened, assessed for eligibility, and  included in the review, with reasons for exclusions at each stage,  ideally with a flow diagram. | 4-5 |
| Presentation of  network structure | S3 | Provide a network graph of the included studies to enable  visualization of the geometry of the treatment network. | 4-5 |
| Summary of  network geometry | S4 | Provide a brief overview of characteristics of the treatment network.  This may include commentary on the abundance of trials and  randomized patients for the different interventions and pairwise  comparisons in the network, gaps of evidence in the treatment  network, and potential biases reflected by the network structure. | 5 |
| Study  characteristics | 18 | For each study, present characteristics for which data were extracted  (e.g., study size, PICOS, follow-up period) and provide the citations. | 5 |
| Risk of bias  within studies | 19 | Present data on risk of bias of each study and, if available, any  outcome level assessment | 5-6 |
| Results of  individual studies | 20 | For all outcomes considered (benefits or harms), present, for each  study: 1) simple summary data for each intervention group, and 2)  effect estimates and confidence intervals. *Modified approaches may*  *be needed to deal with information from larger networks.* | 6-8 |
| Synthesis of  results | 21 | Present results of each meta-analysis done, including  confidence/credible intervals. *In larger networks, authors may focus*  *on comparisons versus a particular comparator (e.g., placebo or*  *standard care), with full findings presented in an appendix. League*  *tables and forest plots may be considered to summarize pairwise*  *comparisons.* If additional summary measures were explored (such  as treatment rankings), these should also be presented. | 6-8 |
| Exploration for  inconsistency | S5 | Describe results from investigations of inconsistency. This may  include such information as measures of model fit to compare  consistency and inconsistency models, *P* values from statistical  tests, or summary of inconsistency estimates from different parts of  the treatment network. | 8-9 |
| Risk of bias  across studies | 22 | Present results of any assessment of risk of bias across studies for  the evidence base being studied. | 9-10 |
| Results of  additional  analyses | 23 | Give results of additional analyses, if done (e.g., sensitivity or  subgroup analyses, meta-regression analyses*, alternative network*  *geometries studied, alternative choice of prior distributions for*  *Bayesian analyses,* and so forth). | 11 |
| **DISCUSSION** | | | |
| Summary of  evidence | 24 | Summarize the main findings, and the mechanisms by which various intervention measures achieve therapeutic efficacy are extensively discussed, alongside unresolved issues in current research. | 12-15 |
| Limitations | 25 | Discuss limitations at study and outcome level (e.g., risk of bias),  and at review level (e.g., incomplete retrieval of identified research,  reporting bias). *Comment on the validity of the assumptions, such as*  *transitivity and consistency. Comment on any concerns regarding*  *network geometry (e.g., avoidance of certain comparisons).* | 16 |
| Conclusions | 26 | Provide a general interpretation of the results in the context of other  evidence, and implications for future research. | 16 |

PICOS = population, intervention, comparators, outcomes, study design.

* Text in italics indicate wording specific to reporting of network meta-analyses that has been added to guidance from the PRISMA statement.

† Authors may wish to plan for use of appendices to present all relevant information in full detail for items in this section.

| Author | year | Intervention  **Supplemental Table 2 Characteristics of the included studies** | Control | Sample | Age | Sex（M/F） | Follow up (weeks) | Outcome Indicators(pain) | Outcome Indicators(function) |
| --- | --- | --- | --- | --- | --- | --- | --- | --- | --- |
| Çağlar, Yağcı H^[1]^ | 2023 | ESWT:  2000 impulses at 2 bar at 12Hz  3 sessions (weekly interval) | CSI:1 mL of 40 mg triamcinolone acetonide and 2 mL of 2% prilocaine hydrochloride (20 mg/mL) | I：32 | I: 50.0±9.1 | I:(5/27) | 0,3,12w | VAS | / |
|  |  |  |  | C：28 | C: 51.7±7.7 | C:(7/21) |  |  |  |
| Heaver, C^[2]^ | 2023 | ESWT:  2500 impulses at 0.15–0.35mJ/mm2  3 sessions (weekly interval) | CSI:80mg of Depo  Medrone (methylprednisolone) with 3.5ml 0.5% bupivic  aine and 3.5ml 1% lignocaine | I：48 | I: 63.73±11.87 | I:(6/42) | 0,12,48w | VAS | HHS |
|  |  |  |  | C：47 | C: 60.31±12.74 | C:(4/43) |  |  |  |
| Notarnicola, A^[3]^ | 2023 | ESWT:  2000 impulses at 0.03–0.17mJ/mm2 at 4 Hz  3 sessions (weekly interval) | EX:eccentric therapeutic exercise for 5 days a week and a total of 4 weeks (Piriformis muscle stretching ; gluteal muscle stretching ; wall squat with a ball ; leg lift ; iliotibial band stretching) | I：15 | I: 59.1±9.6 | I:(3/12) | 0,8,16,24w | NRS | / |
|  |  |  |  | C：15 | C: 59.5±7.7 | C:(2/13) |  |  |  |
| Wheeler, P. C^[4]^ | 2022 | ESWT:  2000 impulses at 20 Hz  3 sessions (weekly interval) | CON:  ESWT 500 impulses  3 sessions (weekly interval) | I：57 | I: 59.1±9.6 | I:(8/49) | 0,6,12,24w | NRS | VISA-G |
|  |  |  |  | C：63 | C: 59.5±7.7 | C:(15/48) |  |  |  |
| Mellor, R^[5]^ | 2022 | EX：  Exercise programme of targeted strengthening of the hip abductor muscles and dynamic control of adduction during function (4–6 exercises to be performed daily)  CSI：  (1mL Celestone (betamethasone5.7mg/mL) or 1mL Kenacort A40(triamcinolone acetonide 40mg/mL) and local anaesthetic (2mL bupivacaine or 1mL Marcaine) | CON：  Wait and see | I1：69 | I1: 54.8±8.1 | I1:(13/56) | 0,4,8,12,26,52w | NRS | / |
|  |  |  |  | I2：66 | I2: 55.3±9.4 | I2:(9/57) |  |  |  |
|  |  |  |  | C：69 | C: 54.5±9.1 | C:(15/54) |  |  |  |
| Rosário, D^[6]^ | 2021 | BMAC:Using an ultrasound-guided standard 10-mL syringe, 20 mL of the solution with BMAC was injected in the gluteal tendon footprint | CSI:7 mg of betamethasone associated with 4 mL of 2% lidocaine without vasoconstrictor | I：15 | I: 46.1±15.2 | I:(10/5) | 0,4,24w | VAS | / |
|  |  |  |  | C：25 | C: 53.2±12.0 | C:(10/15) |  |  |  |
| Ramon, S^[7]^ | 2020 | ESWT:  2000 impulses at 0.20mJ/mm2 at 5Hz  3 sessions (weekly interval) | CON:  2000 impulses at 0.01mJ/mm2 (the lowest EFD of the device) at 5Hz  3 sessions (weekly interval) | I：53 | I: 57.1±12.9 | I:(9/42) | 0,4,8,12,24w | VAS | HHS |
|  |  |  |  | C：50 | C: 55.6±11 | C:(18/32) |  |  |  |
| Begkas, D^[8]^ | 2020 | PRP:  40 ml of autologous blood was taken from the antecubital vein, and 4 ml of PRP were withdrawn | CSI:  a single injection of 4 ml of methylprednisolone (40 mg/ml) | I：12 | \ | I:(2/10) | 0,4,12,24w | VAS | HHS |
|  |  |  |  | C：12 |  | C:(4/8) |  |  |  |
| Clifford, C^[9]^ | 2019 | EX(Isometric exercise programme):hip abduction hold;weight-bearing gluteal contraction. | EX(Isotonic exercise programme): side-lying hip abduction;hip abduction slide | I：15 | I: 57.5±16.8 | I:(2/13) | 0,4,12w | NRS | VISA-G |
|  |  |  |  | C：15 | C:61.1±15.2 | C:(1/14) |  |  |  |
| Thompson, G.^[10]^ | 2019 | PRP:  Five millilitres of PRP was added to 1ml 1% xylocaine | CON:  5mls isotonic saline was added to the 1ml 1% xylocaine | I：24 | I: 54.3±10.5 | I:(2/22) | 12,24,48w | NRS | / |
|  |  |  |  | C：24 | C: 56.3±9.6 | C:(0/24) |  |  |  |
| Carlisi, E.^[11]^ | 2019 | ESWT:  1800 impulses at 3 bar at 0.15mJ/mm2  3 sessions (weekly interval) | UST:  an intensity of 1.5W/cm2 in sessions of  10minutes each(ROLAND, RT-20 series, frequency=1MHz) | I：26 | I: 61±9.18 | I:(5/21) | 0,8,24w | NRS | / |
|  |  |  |  | C：24 | C: 61.5±9.52 | C:(2/22) |  |  |  |
| Fitzpatrick, J.^[12]^ | 2019 | PRP:  Volume of blood taken/additives 52 mL, blood; ACD-A, 8 mL;  Volume delivered 6-7 mL | CSI | I：40 | I: 59.7 | I:(2/48) | 0,2,6,12,24,  52,104w | / | HHS |
|  |  |  |  | C：40 | C: 60.3 | C:(6/34) |  |  |  |
| Ganderton, C^[13]^ | 2018 | EX:  GLoBE exercise program stages 1–4: hip hitch; double leg wall squat;double leg calf raises;Hip hitch with toe tap;sit to stand; calf raises with toe taps; hip hitch with hip swing; sit to stand with split stance; single leg calf raises; single leg squat;step up;single leg calf raise. | CON:  Sham exercise protocol(seated exercises not aimed at therapeutic loading of the gluteal tendons or strengthening of the kinetic chain) | I：46 | I: 61.14±6.70 | / | 0,12,52w | NRS | VISA-G |
|  |  |  |  | C：48 | C: 62.53±8.92 |  |  |  |  |
| Mellor, R^[14]^ | 2018 | EX：  Exercise programme of targeted strengthening of the hip abductor muscles and dynamic control of adduction during function (4–6 exercises to be performed daily)  CSI：  (1mL Celestone (betamethasone5.7mg/mL) or 1mL Kenacort A40(triamcinolone acetonide 40mg/mL) and local anaesthetic (2mL bupivacaine or 1mL Marcaine) | CON：  Wait and see | I1：69 | I1: 54.8±8.1 | I1:(13/56) | 0,4,8,12,26,52w | NRS | / |
|  |  |  |  | I2：66 | I2: 55.3±9.4 | I2:(9/57) |  |  |  |
|  |  |  |  | C：69 | C: 54.5±9.1 | C:(15/54) |  |  |  |
| Fitzpatrick, J.^[15]^ | 2018 | PRP:  Volume of blood taken/additives 52 mL, blood; ACD-A, 8 mL;  Volume delivered 6-7 mL | CSI | I：40 | I: 59.7 | I:(2/48) | 0,2,6,12w | / | HHS |
|  |  |  |  | C：40 | C: 60.3 | C:(6/34) |  |  |  |
| Acosta Pereira,A^[16]^ | 2017 | HA:  intra-bursal injection of: 40 mg triamcinolone acetonide (Trigon Depot) plus 1mL lidocaine | CSI:  40 mg triamcinolone acetonide (Trigon Depot) plus 60 mg HA depot | I：25 | I: 63.95±12.0 | / | 0,4,12,24w | VAS | / |
|  |  |  |  | C：22 | C: 67.83±11.1 |  |  |  |  |
| Brennan, K L^[17]^ | 2017 | DN:  needle length typically ranged from 50 to 100 mm, with a diameter of 0.30 to 0.50 mm,the needle then was left in situ for approximately 5 to 7 minutes | CSI:  2 mL methylprednisolone acetate (Depo-Medrol; Pfizer Inc, New York, NY), 40 mg/mL; 4 mL 1% lidocaine; 4 mL 0.25% marcaine (10 mL total) | I：21 | I: 61.3±16.5 | I:(2/19) | 0,1,3,6w | NRS | / |
|  |  |  |  | C：22 | C: 70.1±11.4 | C:(4/18) |  |  |  |
| Brinks, A^[18]^ | 2011 | CSI:  40 mg of triamcinolone acetate combined with 1% or 2% lidocaine in a 5-mL syringe | CON:  usual care | I：60 | I: 54.8±14.7 | I:(12/48) | 0,12,48w | NRS | / |
|  |  |  |  | C：60 | C: 57.7±13.9 | C:(16/44) |  |  |  |
| Rompe, J. D.^[19]^ | 2009 | EX  ESWT:2000 impulses at 3 bar at 0.12mJ/mm2  3 sessions (weekly interval) | CSI:  5 mL of 0.5% Mepivacain (Meaverin 0.5%, DeltaSelect  GmbH, Dreieich, Germany) mixed with 1 mL of Prednisolone  (25 mg, Predni 25 mg Lichtenstein N Kristallsuspension,  Winthrop Arzneimittel GmbH, Fürstenfeldbruck, Germany). | I1：76 | I1: 46 | I1:(23/53) | 0,4,16,60w | NRS | / |
|  |  |  |  | I2：75 | I2: 50 | I2:(2/54) |  |  |  |
|  |  |  |  | C：78 | C: 47 | C:(23/55) |  |  |  |

EX: exercise, CSI:corticosteroid injection, BMAC:bone marrow aspirate concentrate, HA: hyaluronic acid，ESWT:extracorporeal shockwave therapy, PRP: platelet-rich plasma injection, DN: dry needling, UST:ultrasound therapy, CON: control(sham exercise or placebo), VAS: visual analogue scale, NRS: numeric rating sclae, VISA-G: Victoria Institute of Sport—Gluteal score, HHS:Harris Hip Score

|  | **Pubmed 522 results (MESH Terms)**  **Supplemental Table 3**  Literature Search Strategies for PubMed, Web of Science, Cochrane Library, and EMBASE | **Embase 422 results (Emtree Terms)** | **Cochrane 128 results (MESH Terms)** | **Web of science 434 results** |
| --- | --- | --- | --- | --- |
| **GTPS**  **#1** | ((((((greater trochanter pain syndrome ) OR (GTPS )) OR (Trochanteric bursitis )) OR (Gluteal tendinopathy )) OR (Gluteus medius tendinopathy )) OR (Gluteus minimus tendinopathy)) OR (greater trochanteric pain syndrome) | 'greater trochanter pain syndrome' OR 'gtps' OR 'trochanteric bursitis' OR 'gluteal tendinopathy' OR 'gluteus medius tendinopathy' OR 'gluteus minimus tendinopathy' OR 'greater trochanteric pain syndrome' | (greater trochanter pain syndrome) OR (GTPS) OR (Trochanteric bursitis) OR (Gluteal tendinopathy) OR (Gluteus Medius tendinopathy) OR (gluteus Minimus tendinosis) OR (greater trochanteric pain syndrome) | (TS=((greater trochanter pain syndrome OR GTPS OR Trochanteric bursitis OR Gluteal tendinopathy OR Gluteus medius tendinopathy OR Gluteus minimus tendinopathy OR greater trochanteric pain syndrome) )) NOT (SILOID==("PPRN")) |
| **Exercise**  **#2** | (((((((((((((((((((((((((Exercise) OR (Exercises)) OR (Physical Activity)) OR (Activities, Physical)) OR (Activity, Physical)) OR (Physical Activities)) OR (Exercise, Physical)) OR (Exercises, Physical)) OR (Physical Exercise)) OR (Physical Exercises)) OR (Acute Exercise)) OR (Acute Exercises )) OR (Exercise, Acute)) OR (Exercises, Acute)) OR (Exercise, Isometric)) OR (Exercises, Isometric)) OR (Isometric Exercises)) OR (Isometric Exercise)) OR (Exercise, Aerobic)) OR (Aerobic Exercise)) OR (Aerobic Exercises)) OR (Exercises, Aerobic)) OR (Exercise Training)) OR (Exercise Trainings)) OR (Training, Exercise)) OR (Trainings, Exercise) | 'exercise' OR 'exercises' OR 'physical activity' OR 'activities, physical' OR 'activity, physical' OR 'physical activities' OR 'exercise, physical' OR 'exercises, physical' OR 'physical exercise' OR 'physical exercises' OR 'acute exercise' OR 'acute exercises' OR 'exercise, acute' OR 'exercises, acute' OR 'exercise, isometric' OR 'exercises, isometric' OR 'isometric exercises' OR 'isometric exercise' OR 'exercise, aerobic' OR 'aerobic exercise' OR 'aerobic exercises' OR 'exercises, aerobic' OR 'exercise training' OR 'exercise trainings' OR 'training, exercise' OR 'trainings, exercise' | (Exercise) OR (Exercises) OR (Physical Activity) OR (Activities, Physical) OR (Activity, Physical) OR (Physical Activities) OR (Exercise, Physical) OR (Exercises, Physical) OR (Physical Exercise) OR (Physical Exercises) OR (Acute Exercise) OR (Acute Exercises) OR (Exercise, Acute) OR (Exercises, Acute) OR (Exercise, Isometric) OR (Exercises, Isometric) OR (Isometric Exercises) OR (Isometric Exercise) OR (Exercise, Aerobic) OR (Aerobic Exercise) OR (Aerobic Exercises) OR (Exercises, Aerobic) OR (Exercise Training) OR (Exercise Trainings) OR (Training, Exercise) OR (Trainings, Exercise) | TS=((Exercise OR Exercises OR Physical Activity OR Activities, Physical OR Activity, Physical OR Physical Activities OR Exercise, Physical OR Exercises, Physical OR Physical Exercise OR Physical Exercises OR Acute Exercise OR Acute Exercises OR Exercise, Acute OR Exercises, Acute OR Exercise, Isometric OR Exercises, Isometric OR Isometric Exercises OR Isometric Exercise OR Exercise, Aerobic OR Aerobic Exercise OR Aerobic Exercises OR Exercises, Aerobic OR Exercise Training OR Exercise Trainings OR Training, Exercise OR Trainings, Exercise) ) and Preprint Citation Index (Exclude – Database) |
| **ESWT**  **#3** | (((((((((((((((Extracorporeal Shockwave Therapy) OR (Extracorporeal Shockwave Therapies)) OR (Shockwave Therapies, Extracorporeal)) OR (Shockwave Therapy, Extracorporeal)) OR (Therapy, Extracorporeal Shockwave)) OR (Shock Wave Therapy)) OR (Shock Wave Therapies)) OR (Therapy, Shock Wave)) OR (Extracorporeal Shock Wave Therapy)) OR (Extracorporeal High-Intensity Focused Ultrasound Therapy)) OR (Extracorporeal High Intensity Focused Ultrasound Therapy)) OR (HIFU Therapy)) OR (HIFU Therapies)) OR (Therapy, HIFU)) OR (High-Intensity Focused Ultrasound Therapy)) OR (High Intensity Focused Ultrasound Therapy) | 'extracorporeal shockwave therapy' OR 'extracorporeal shockwave therapies' OR 'shockwave therapies, extracorporeal' OR 'shockwave therapy, extracorporeal' OR 'therapy, extracorporeal shockwave' OR 'shock wave therapy' OR 'shock wave therapies' OR 'therapy, shock wave' OR 'extracorporeal shock wave therapy' OR 'extracorporeal high-intensity focused ultrasound therapy' OR 'extracorporeal high intensity focused ultrasound therapy' OR 'hifu therapy' OR 'hifu therapies' OR 'therapy, hifu' OR 'high-intensity focused ultrasound therapy' OR 'high intensity focused ultrasound therapy' | (Extracorporeal Shockwave Therapy) OR (Extracorporeal Shockwave Therapies) OR (Shockwave Therapies, Extracorporeal) OR (Shockwave Therapy, Extracorporeal) OR (Therapy, Extracorporeal Shockwave) OR (Shock Wave Therapy) OR (Shock Wave Therapies) OR (Therapy, Shock Wave) OR (Extracorporeal Shock Wave Therapy) OR (Extracorporeal High-Intensity Focused Ultrasound Therapy) OR (Extracorporeal High Intensity Focused Ultrasound Therapy) OR (HIFU Therapy) OR (HIFU Therapies) OR (Therapy, HIFU) OR (High-Intensity Focused Ultrasound Therapy) OR (High Intensity Focused Ultrasound Therapy) | TS=((Extracorporeal Shockwave Therapy OR Extracorporeal Shockwave Therapies OR Shockwave Therapies, Extracorporeal OR Shockwave Therapy, Extracorporeal OR Therapy, Extracorporeal Shockwave OR Shock Wave Therapy OR Shock Wave Therapies OR Therapy, Shock Wave OR Extracorporeal Shock Wave Therapy OR Extracorporeal High-Intensity Focused Ultrasound Therapy OR Extracorporeal High Intensity Focused Ultrasound Therapy OR HIFU Therapy OR HIFU Therapies OR Therapy, HIFU OR High-Intensity Focused Ultrasound Therapy OR High Intensity Focused Ultrasound Therapy)) and Preprint Citation Index (Exclude – Database) |
| **Physical Therapy**  **#4** | ((((((((((((((((((Physical Therapy Modalities) OR (Modalities, Physical Therapy)) OR (Modality, Physical Therapy)) OR (Physical Therapy Modality)) OR (Physiotherapy (Techniques))) OR (Physiotherapies (Techniques))) OR (Physical Therapy Techniques)) OR (Physical Therapy Technique)) OR (Techniques, Physical Therapy)) OR (Group Physiotherapy)) OR (Group Physiotherapies)) OR (Physiotherapies, Group)) OR (Physiotherapy, Group)) OR (Physical Therapy)) OR (Physical Therapies)) OR (Therapy, Physical)) OR (Neurological Physiotherapy)) OR (Physiotherapy, Neurological)) OR (Neurophysiotherapy) | 'physical therapy modalities' OR 'modalities, physical therapy' OR 'modality, physical therapy' OR 'physical therapy modality' OR 'physiotherapy (techniques)' OR 'physiotherapies (techniques)' OR 'physical therapy techniques' OR 'physical therapy technique' OR 'techniques, physical therapy' OR 'group physiotherapy' OR 'group physiotherapies' OR 'physiotherapies, group' OR 'physiotherapy, group' OR 'physical therapy' OR 'physical therapies' OR 'therapy, physical' OR 'neurological physiotherapy' OR 'physiotherapy, neurological' OR 'neurophysiotherapy' | (Physical Therapy Modalities) OR (Modalities, Physical Therapy) OR (Modality, Physical Therapy) OR (Physical Therapy Modality) OR (Physiotherapy (Techniques)) OR (Physiotherapies (Techniques)) OR (Physical Therapy Techniques) OR (Physical Therapy Technique) OR (Techniques, Physical Therapy) OR (Group Physiotherapy) OR (Group Physiotherapies) OR (Physiotherapies, Group) OR (Physiotherapy, Group) OR (Physical Therapy) OR (Physical Therapies) OR (Therapy, Physical) OR (Neurological Physiotherapy) OR (Physiotherapy, Neurological) OR (Neurophysiotherapy) | TS=((Physical Therapy Modalities OR Modalities, Physical Therapy OR Modality, Physical Therapy OR Physical Therapy Modality OR Physiotherapy (Techniques) OR Physiotherapies (Techniques) OR Physical Therapy Techniques OR Physical Therapy Technique OR Techniques, Physical Therapy OR Group Physiotherapy OR Group Physiotherapies OR Physiotherapies, Group OR Physiotherapy, Group OR Physical Therapy OR Physical Therapies OR Therapy, Physical OR Neurological Physiotherapy OR Physiotherapy, Neurological OR Neurophysiotherapy)) and Preprint Citation Index (Exclude – Database) |
| **PRP**  **#5** | ((Platelet-Rich Plasma) OR (Plasma, Platelet-Rich)) OR (Platelet Rich Plasma) | 'platelet-rich plasma' OR 'plasma, platelet-rich' OR 'platelet rich plasma' | (Platelet-Rich Plasma) OR (Plasma, Platelet-Rich) OR (Platelet Rich Plasma) | TS=((Platelet-Rich Plasma OR Plasma, Platelet-Rich OR Platelet Rich Plasma)) and Preprint Citation Index (Exclude – Database) |
| **Stem cells**  **#6** | (((((((((((((((Stem Cells) OR (Cell, Stem)) OR (Cells, Stem)) OR (Stem Cell)) OR (Progenitor Cells)) OR (Cell, Progenitor)) OR (Cells, Progenitor)) OR (Progenitor Cell)) OR (Mother Cells)) OR (Cell, Mother)) OR (Cells, Mother)) OR (Mother Cell)) OR (Colony-Forming Unit)) OR (Colony Forming Unit)) OR (Colony-Forming Units)) OR (Colony Forming Units) | 'stem cells' OR 'cell, stem' OR 'cells, stem' OR 'stem cell' OR 'progenitor cells' OR 'cell, progenitor' OR 'cells, progenitor' OR 'progenitor cell' OR 'mother cells' OR 'cell, mother' OR 'cells, mother' OR 'mother cell' OR 'colony-forming unit' OR 'colony forming unit' OR 'colony-forming units' OR 'colony forming units'90 | (Stem Cells) OR (Cell, Stem) OR (Cells, Stem) OR (Stem Cell) OR (Progenitor Cells) OR (Cell, Progenitor) OR (Cells, Progenitor) OR (Progenitor Cell) OR (Mother Cells) OR (Cell, Mother) OR (Cells, Mother) OR (Mother Cell) OR (Colony-Forming Unit) OR (Colony Forming Unit) OR (Colony-Forming Units) OR (Colony Forming Units) | TS=((Stem Cells OR Cell, Stem OR Cells, Stem OR Stem Cell OR Progenitor Cells OR Cell, Progenitor OR Cells, Progenitor OR Progenitor Cell OR Mother Cells OR Cell, Mother OR Cells, Mother OR Mother Cell OR Colony-Forming Unit OR Colony Forming Unit OR Colony-Forming Units OR Colony Forming Units)) and Preprint Citation Index (Exclude – Database) |
| **Hydrogels**  **#7** | (((((((Hydrogels) OR (Hydrogel)) OR (In Situ Hydrogels)) OR (In Situ Hydrogel)) OR (Hydrogel, In Situ)) OR (Patterned Hydrogels)) OR (Patterned Hydrogel)) OR (Hydrogel, Patterned) | 'hydrogels' OR 'hydrogel' OR 'in situ hydrogels' OR 'in situ hydrogel' OR 'hydrogel, in situ' OR 'patterned hydrogels' OR 'patterned hydrogel' OR 'hydrogel, patterned' | (Hydrogels) OR (Hydrogel) OR (In Situ Hydrogels) OR (In Situ Hydrogel) OR (Hydrogel, In Situ) OR (Patterned Hydrogels) OR (Patterned Hydrogel) OR (Hydrogel, Patterned) | TS=((Hydrogels OR Hydrogel OR In Situ Hydrogels OR In Situ Hydrogel OR Hydrogel, In Situ OR Patterned Hydrogels OR Patterned Hydrogel OR Hydrogel, Patterned)) and Preprint Citation Index (Exclude – Database) |
| **Glucocorticoids**  **#8** | (((((Glucocorticoids) OR (Glucocorticoid)) OR (Glucocorticoid Effect)) OR (Effect, Glucocorticoid)) OR (Glucorticoid Effects)) OR (Effects, Glucorticoid) | 'glucocorticoids' OR 'glucocorticoid' OR 'glucocorticoid effect' OR 'effect, glucocorticoid' OR 'glucorticoid effects' OR 'effects, glucorticoid' | (Glucocorticoids) OR (Glucocorticoid) OR (Glucocorticoid Effect) OR (Effect, Glucocorticoid) OR (Glucorticoid Effects) OR (Effects, Glucorticoid) | TS=((Glucocorticoids OR Glucocorticoid OR Glucocorticoid Effect OR Effect, Glucocorticoid OR Glucorticoid Effects OR Effects, Glucorticoid)) and Preprint Citation Index (Exclude – Database) |
| **Triamcinolone**  **#9** | ((Triamcinolone) OR (Volon)) OR (Aristocort) | 'triamcinolone' OR 'volon' OR 'aristocort' | (Triamcinolone) OR (Volon) OR (Aristocort) | TS=((Triamcinolone OR Volon OR Aristocort)) and Preprint Citation Index (Exclude – Database) |
| **Corticosteroid**  **#10** | Corticosteroid | 'corticosteroid' | Corticosteroid | TS=(Corticosteroid) and Preprint Citation Index (Exclude – Database) |
| **Magnetic**  **Field Therapy**  **#11** | ((((((((((((((((((((Magnetic Field Therapy) OR (Magnetic Field Therapies)) OR (Therapies, Magnetic Field)) OR (Therapy, Magnetic Field)) OR (Electrically-Charged Magnetic Therapy)) OR (Electrically Charged Magnetic Therapy)) OR (Electrically-Charged Magnetic Therapies)) OR (Magnetic Therapies, Electrically-Charged)) OR (Magnetic Therapy, Electrically-Charged)) OR (Therapy, Electrically-Charged Magnetic)) OR (Magnetic Stimulation Therapy)) OR (Magnetic Stimulation Therapies)) OR (Stimulation Therapy, Magnetic)) OR (Therapies, Magnetic Stimulation)) OR (Therapy, Magnetic Stimulation)) OR (Magnetotherapy)) OR (Static Magnetic Field Therapy)) OR (Electromagnetic Therapy)) OR (Electromagnetic Therapies)) OR (Therapies, Electromagnetic)) OR (Therapy, Electromagnetic) | 'magnetic field therapy' OR 'magnetic field therapies' OR 'therapies, magnetic field' OR 'therapy, magnetic field' OR 'electrically-charged magnetic therapy' OR 'electrically charged magnetic therapy' OR 'electrically-charged magnetic therapies' OR 'magnetic therapies, electrically-charged' OR 'magnetic therapy, electrically-charged' OR 'therapy, electrically-charged magnetic' OR 'magnetic stimulation therapy' OR 'magnetic stimulation therapies' OR 'stimulation therapy, magnetic' OR 'therapies, magnetic stimulation' OR 'therapy, magnetic stimulation' OR 'magnetotherapy' OR 'static magnetic field therapy' OR 'electromagnetic therapy' OR 'electromagnetic therapies' OR 'therapies, electromagnetic' OR 'therapy, electromagnetic' | (Magnetic Field Therapy) OR (Magnetic Field Therapies) OR (Therapies, Magnetic Field) OR (Therapy, Magnetic Field) OR (Electrically-Charged Magnetic Therapy) OR (Electrically Charged Magnetic Therapy) OR (Electrically-Charged Magnetic Therapies) OR (Magnetic Therapies, Electrically-Charged) OR (Magnetic Therapy, Electrically-Charged) OR (Therapy, Electrically-Charged Magnetic) OR (Magnetic Stimulation Therapy) OR (Magnetic Stimulation Therapies) OR (Stimulation Therapy, Magnetic) OR (Therapies, Magnetic Stimulation) OR (Therapy, Magnetic Stimulation) OR (Magnetotherapy) OR (Static Magnetic Field Therapy) OR (Electromagnetic Therapy) OR (Electromagnetic Therapies) OR (Therapies, Electromagnetic) OR (Therapy, Electromagnetic) | TS=((Magnetic Field Therapy OR Magnetic Field Therapies OR Therapies, Magnetic Field OR Therapy, Magnetic Field OR Electrically-Charged Magnetic Therapy OR Electrically Charged Magnetic Therapy OR Electrically-Charged Magnetic Therapies OR Magnetic Therapies, Electrically-Charged OR Magnetic Therapy, Electrically-Charged OR Therapy, Electrically-Charged Magnetic OR Magnetic Stimulation Therapy OR Magnetic Stimulation Therapies OR Stimulation Therapy, Magnetic OR Therapies, Magnetic Stimulation OR Therapy, Magnetic Stimulation OR Magnetotherapy OR Static Magnetic Field Therapy OR Electromagnetic Therapy OR Electromagnetic Therapies OR Therapies, Electromagnetic OR Therapy, Electromagnetic)) and Preprint Citation Index (Exclude – Database) |
| **Hyaluronic Acid**  **#12** | (((((((((((((Hyaluronic Acid) OR (Acid, Hyaluronic)) OR (Amo Vitrax)) OR (Vitrax, Amo)) OR (Biolon)) OR (Etamucine)) OR (Hyaluronan)) OR (Hyvisc)) OR (Luronit)) OR (Sodium Hyaluronate)) OR (Hyaluronate, Sodium)) OR (Hyaluronate Sodium)) OR (Amvisc)) OR (Healon) | 'hyaluronic acid' OR 'acid, hyaluronic' OR 'amo vitrax' OR 'vitrax, amo' OR 'biolon' OR 'etamucine' OR 'hyaluronan' OR 'hyvisc' OR 'luronit' OR 'sodium hyaluronate' OR 'hyaluronate, sodium' OR 'hyaluronate sodium' OR 'amvisc' OR 'healon' | (Hyaluronic Acid) OR (Acid, Hyaluronic) OR (Amo Vitrax) OR (Vitrax, Amo) OR (Biolon) OR (Etamucine) OR (Hyaluronan) OR (Hyvisc) OR (Luronit) OR (Sodium Hyaluronate) OR (Hyaluronate, Sodium) OR (Hyaluronate Sodium) OR (Amvisc) OR (Healon) | TS=((Hyaluronic Acid OR Acid, Hyaluronic OR Amo Vitrax OR Vitrax, Amo OR Biolon OR Etamucine OR Hyaluronan OR Hyvisc OR Luronit OR Sodium Hyaluronate OR Hyaluronate, Sodium OR Hyaluronate Sodium OR Amvisc OR Healon)) and Preprint Citation Index (Exclude – Database) |
| **Low-Level Light Therapy**  **#13** | ((((((((((((((((((((((((((((((((((Low-Level Light Therapy) OR (Light Therapies, Low-Level)) OR (Light Therapy, Low-Level)) OR (Low Level Light Therapy)) OR (Low-Level Light Therapies)) OR (Therapies, Low-Level Light)) OR (Therapy, Low-Level Light)) OR (Photobiomodulation Therapy)) OR (Photobiomodulation Therapies)) OR (Therapies, Photobiomodulation)) OR (Therapy, Photobiomodulation)) OR (Photobiomodulation)) OR (Photobiomodulations)) OR (LLLT)) OR (Laser Therapy, Low-Level)) OR (Laser Therapies, Low-Level)) OR (Laser Therapy, Low Level)) OR (Low-Level Laser Therapies)) OR (Laser Irradiation, Low-Power)) OR (Irradiation, Low-Power Laser)) OR (Laser Irradiation, Low Power)) OR (Low-Power Laser Therapy)) OR (Low Power Laser Therapy)) OR (Laser Therapy, Low-Power)) OR (Laser Therapies, Low-Power)) OR (Laser Therapy, Low Power)) OR (Low-Power Laser Therapies)) OR (Low-Level Laser Therapy)) OR (Low Level Laser Therapy)) OR (Low-Power Laser Irradiation)) OR (Low Power Laser Irradiation)) OR (Laser Biostimulation)) OR (Biostimulation, Laser)) OR (Laser Phototherapy)) OR (Phototherapy, Laser) | 'low-level light therapy' OR 'light therapies, low-level' OR 'light therapy, low-level' OR 'low level light therapy' OR 'low-level light therapies' OR 'therapies, low-level light' OR 'therapy, low-level light' OR 'photobiomodulation therapy' OR 'photobiomodulation therapies' OR 'therapies, photobiomodulation' OR 'therapy, photobiomodulation' OR 'photobiomodulation' OR 'photobiomodulations' OR 'lllt' OR 'laser therapy, low-level' OR 'laser therapies, low-level' OR 'laser therapy, low level' OR 'low-level laser therapies' OR 'laser irradiation, low-power' OR 'irradiation, low-power laser' OR 'laser irradiation, low power' OR 'low-power laser therapy' OR 'low power laser therapy' OR 'laser therapy, low-power' OR 'laser therapies, low-power' OR 'laser therapy, low power' OR 'low-power laser therapies' OR 'low-level laser therapy' OR 'low level laser therapy' OR 'low-power laser irradiation' OR 'low power laser irradiation' OR 'laser biostimulation' OR 'biostimulation, laser' OR 'laser phototherapy' OR 'phototherapy, laser' | (Low-Level Light Therapy) OR (Light Therapies, Low-Level) OR (Light Therapy, Low-Level) OR (Low Level Light Therapy) OR (Low-Level Light Therapies) OR (Therapies, Low-Level Light) OR (Therapy, Low-Level Light) OR (Photobiomodulation Therapy) OR (Photobiomodulation Therapies) OR (Therapies, Photobiomodulation) OR (Therapy, Photobiomodulation) OR (Photobiomodulation) OR (Photobiomodulations) OR (LLLT) OR (Laser Therapy, Low-Level) OR(Laser Therapies, Low-Level) OR (Laser Therapy, Low Level) OR (Low-Level Laser Therapies) OR (Laser Irradiation, Low-Power) OR (Irradiation, Low-Power Laser) OR (Laser Irradiation, Low Power) OR (Low-Power Laser Therapy) OR (Low Power Laser Therapy) OR (Laser Therapy, Low-Power) OR (Laser Therapies, Low-Power) OR (Laser Therapy, Low Power) OR (Low-Power Laser Therapies) OR (Low-Level Laser Therapy) OR (Low Level Laser Therapy) OR (Low-Power Laser Irradiation) OR (Low Power Laser Irradiation) OR (Laser Biostimulation) OR (Biostimulation, Laser) OR (Laser Phototherapy) OR (Phototherapy, Laser) | TS=((Low-Level Light Therapy OR Light Therapies, Low-Level OR Light Therapy, Low-Level OR Low Level Light Therapy OR Low-Level Light Therapies OR Therapies, Low-Level Light OR Therapy, Low-Level Light OR Photobiomodulation Therapy OR Photobiomodulation Therapies OR Therapies, Photobiomodulation OR Therapy, Photobiomodulation OR Photobiomodulation OR Photobiomodulations OR LLLT OR Laser Therapy, Low-Level OR Laser Therapies, Low-Level OR Laser Therapy, Low Level OR Low-Level Laser Therapies OR Laser Irradiation, Low-Power OR Irradiation, Low-Power Laser OR Laser Irradiation, Low Power OR Low-Power Laser Therapy OR Low Power Laser Therapy OR Laser Therapy, Low-Power OR Laser Therapies, Low-Power OR Laser Therapy, Low Power OR Low-Power Laser Therapies OR Low-Level Laser Therapy OR Low Level Laser Therapy OR Low-Power Laser Irradiation OR Low Power Laser Irradiation OR Laser Biostimulation OR Biostimulation, Laser OR Laser Phototherapy OR Phototherapy, Laser)) and Preprint Citation Index (Exclude – Database) |
| **Ultrasonic Therapy**  **#14** | ((((((((Ultrasonic Therapy) OR (herapies, Ultrasonic)) OR (Therapeutic Ultrasound)) OR (Ultrasound, Therapeutic)) OR (Therapy, Ultrasonic)) OR (Ultrasound Therapy)) OR (Therapies, Ultrasound)) OR (Therapy, Ultrasound)) OR (Ultrasound Therapies) | 'ultrasonic therapy' OR 'herapies, ultrasonic' OR 'therapeutic ultrasound' OR 'ultrasound, therapeutic' OR 'therapy, ultrasonic' OR 'ultrasound therapy' OR 'therapies, ultrasound' OR 'therapy, ultrasound' OR 'ultrasound therapies' | (Ultrasonic Therapy) OR (herapies, Ultrasonic) OR (Therapeutic Ultrasound) OR (Ultrasound, Therapeutic) OR (Therapy, Ultrasonic) OR (Ultrasound Therapy) OR (Therapies, Ultrasound) OR (Therapy, Ultrasound) OR (Ultrasound Therapies) | TS=((Ultrasonic Therapy OR herapies, Ultrasonic OR Therapeutic Ultrasound OR Ultrasound, Therapeutic OR Therapy, Ultrasonic OR Ultrasound Therapy OR Therapies, Ultrasound OR Therapy, Ultrasound OR Ultrasound Therapies)) and Preprint Citation Index (Exclude – Database) |
| **Dry Needling**  **#15** | (Dry Needling) OR (Needling, Dry) | 'dry needling' OR 'needling, dry' | (Dry Needling) OR (Needling, Dry) | TS=((Dry Needling OR Needling, Dry)) and Preprint Citation Index (Exclude – Database) |
| **Fever**  **Therapy**  **#16** | ((((((((Hyperthermia, Induced) OR (Therapy, Fever)) OR (Hyperthermia, Therapeutic)) OR (Induced Hyperthermia)) OR (Therapeutic Hyperthermia)) OR (Thermotherapy)) OR (Fever Therapy)) OR (Hyperthermia, Local)) OR (Local Hyperthermia) | 'hyperthermia, induced' OR 'therapy, fever' OR 'hyperthermia, therapeutic' OR 'induced hyperthermia' OR 'therapeutic hyperthermia' OR 'thermotherapy' OR 'fever therapy' OR 'hyperthermia, local' OR 'local hyperthermia' | (Hyperthermia, Induced) OR (Therapy, Fever) OR (Hyperthermia, Therapeutic) OR (Induced Hyperthermia) OR (Therapeutic Hyperthermia) OR (Thermotherapy) OR (Fever Therapy) OR (Hyperthermia, Local) | TS=((Hyperthermia, Induced OR Therapy, Fever OR Hyperthermia, Therapeutic OR Induced Hyperthermia OR Therapeutic Hyperthermia OR Thermotherapy OR Fever Therapy OR Hyperthermia, Local )) and Preprint Citation Index (Exclude – Database) |
| **Cold**  **Therapy**  **#17** | (((((Cryotherapy) OR (Cryotherapies)) OR (Cold Therapy)) OR (Cold Therapies)) OR (Therapies, Cold)) OR (Therapy, Cold) | 'cryotherapy' OR 'cryotherapies' OR 'cold therapy' OR 'cold therapies' OR 'therapies, cold' OR 'therapy, cold' | (Cryotherapy) OR (Cryotherapies) OR (Cold Therapy) OR (Cold Therapies) OR (Therapies, Cold) OR (Therapy, Cold) | TS=((Cryotherapy OR Cryotherapies OR Cold Therapy OR Cold Therapies OR Therapies, Cold OR Therapy, Cold )) and Preprint Citation Index (Exclude – Database) |
| **Electric Stimulation Therapy**  **#18** | ((((((((((((((Electric Stimulation Therapy) OR (Therapeutic Electrical Stimulation)) OR (Electrical Stimulation, Therapeutic)) OR (Stimulation, Therapeutic Electrical)) OR (Therapeutic Electric Stimulation)) OR (Electric Stimulation, Therapeutic)) OR (Stimulation, Therapeutic Electric)) OR (Electrical Stimulation Therapy)) OR (Stimulation Therapy, Electrical)) OR (Therapy, Electrical Stimulation)) OR (Therapy, Electric Stimulation)) OR (Stimulation Therapy, Electric)) OR (Electrotherapy)) OR (Interferential Current Electrotherapy)) OR (Electrotherapy, Interferential Current) | 'electric stimulation therapy' OR 'therapeutic electrical stimulation' OR 'electrical stimulation, therapeutic' OR 'stimulation, therapeutic electrical' OR 'therapeutic electric stimulation' OR 'electric stimulation, therapeutic' OR 'stimulation, therapeutic electric' OR 'electrical stimulation therapy' OR 'stimulation therapy, electrical' OR 'therapy, electrical stimulation' OR 'therapy, electric stimulation' OR 'stimulation therapy, electric' OR 'electrotherapy' OR 'interferential current electrotherapy' OR 'electrotherapy, interferential current' | (Electric Stimulation Therapy) OR (Therapeutic Electrical Stimulation) OR (Electrical Stimulation, Therapeutic) OR (Stimulation, Therapeutic Electrical) OR (Therapeutic Electric Stimulation) OR (Electric Stimulation, Therapeutic) OR (Stimulation, Therapeutic Electric) OR (Electrical Stimulation Therapy) OR (Stimulation Therapy, Electrical) OR (Therapy, Electrical Stimulation) OR (Therapy, Electric Stimulation) OR (Stimulation Therapy, Electric) OR (Electrotherapy) OR (Interferential Current Electrotherapy) OR (Electrotherapy, Interferential Current) | TS=((Electric Stimulation Therapy OR Therapeutic Electrical Stimulation OR Electrical Stimulation, Therapeutic OR Stimulation, Therapeutic Electrical OR Therapeutic Electric Stimulation OR Electric Stimulation, Therapeutic OR Stimulation, Therapeutic Electric OR Electrical Stimulation Therapy OR Stimulation Therapy, Electrical OR Therapy, Electrical Stimulation OR Therapy, Electric Stimulation OR Stimulation Therapy, Electric OR Electrotherapy OR Interferential Current Electrotherapy OR Electrotherapy, Interferential Current )) and Preprint Citation Index (Exclude – Database) |

**Search: #1 AND (#2 OR #3 OR #4 OR #5 OR #6 OR #7 OR #8 OR #9 OR #10 OR #11 OR #12 OR #13 OR #14 OR #15 OR #16 OR #17 OR #18)**

**Supplement Table 4.** List of included studies**.**

| 1 | çağlar Y H, Yağcı İ, Bağcıer F. Comparison of shock wave therapy and corticosteroid injection in the treatment of greater trochanteric pain syndrome: A single-blind, randomized study[J]. Turk J Phys Med Rehabil,2023,69(2):180-187. |
| --- | --- |
| 2 | Heaver C, Pinches M, Kuiper J H, et al. Greater trochanteric pain syndrome: focused shockwave therapy versus an ultrasound guided injection: a randomised control trial[J]. Hip Int,2023,33(3):490-499. |
| 3 | Notarnicola A, Ladisa I, Lanzilotta P, et al. Shock Waves and Therapeutic Exercise in Greater Trochanteric Pain Syndrome: A Prospective Randomized Clinical Trial with Cross-Over[J]. J Pers Med,2023,13(6). |
| 4 | Wheeler P C, Dudson C, Calver R, et al. Three Sessions of Radial Extracorporeal Shockwave Therapy Gives No Additional Benefit Over "Minimal-Dose" Radial Extracorporeal Shockwave Therapy for Patients With Chronic Greater Trochanteric Pain Syndrome: A Double-Blinded, Randomized, Controlled Trial[J]. Clin J Sport Med,2022,32(1):e7-e18. |
| 5 | Mellor R, Kasza J, Grimaldi A, et al. Mediators and Moderators of Education Plus Exercise on Perceived Improvement in Individuals With Gluteal Tendinopathy: An Exploratory Analysis of a 3-Arm Randomized Trial[J]. J Orthop Sports Phys Ther,2022,52(12):826-836. |
| 6 | Rosário D, Faleiro T B, Franco B, et al. COMPARISON BETWEEN CONCENTRATED BONE MARROW ASPIRATE AND CORTICOID IN GLUTEAL TENDINOPATHY[J]. Acta Ortop Bras,2021,29(1):26-29. |
| 7 | Ramon S, Russo S, Santoboni F, et al. Focused Shockwave Treatment for Greater Trochanteric Pain Syndrome: A Multicenter, Randomized, Controlled Clinical Trial[J]. J Bone Joint Surg Am,2020,102(15):1305-1311. |
| 8 | Begkas D, Chatzopoulos S T, Touzopoulos P, et al. Ultrasound-guided Platelet-rich Plasma Application Versus Corticosteroid Injections for the Treatment of Greater Trochanteric Pain Syndrome: A Prospective Controlled Randomized Comparative Clinical Study[J]. Cureus,2020,12(1):e6583. |
| 9 | Clifford C, Paul L, Syme G, et al. Isometric versus isotonic exercise for greater trochanteric pain syndrome: a randomised controlled pilot study[J]. BMJ Open Sport Exerc Med,2019,5(1):e558. |
| 10 | Thompson G, Pearson J F. No attributable effects of PRP on greater trochanteric pain syndrome[J]. N Z Med J,2019,132(1507):22-32. |
| 11 | Carlisi E, Cecini M, Di Natali G, et al. Focused extracorporeal shock wave therapy for greater trochanteric pain syndrome with gluteal tendinopathy: a randomized controlled trial[J]. Clin Rehabil,2019,33(4):670-680. |
| 12 | Fitzpatrick J, Bulsara M K, O'Donnell J, et al. Leucocyte-Rich Platelet-Rich Plasma Treatment of Gluteus Medius and Minimus Tendinopathy: A Double-Blind Randomized Controlled Trial With 2-Year Follow-up[J]. Am J Sports Med,2019,47(5):1130-1137. |
| 13 | Ganderton C, Semciw A, Cook J, et al. Gluteal Loading Versus Sham Exercises to Improve Pain and Dysfunction in Postmenopausal Women with Greater Trochanteric Pain Syndrome: A Randomized Controlled Trial[J]. J Womens Health (Larchmt),2018,27(6):815-829. |
| 14 | Mellor R, Bennell K, Grimaldi A, et al. Education plus exercise versus corticosteroid injection use versus a wait and see approach on global outcome and pain from gluteal tendinopathy: prospective, single blinded, randomised clinical trial[J]. BMJ,2018,361:k1662. |
| 15 | Fitzpatrick J, Bulsara M K, O'Donnell J, et al. The Effectiveness of Platelet-Rich Plasma Injections in Gluteal Tendinopathy: A Randomized, Double-Blind Controlled Trial Comparing a Single Platelet-Rich Plasma Injection With a Single Corticosteroid Injection[J]. Am J Sports Med,2018,46(4):933-939. |
| 16 | Brennan K L, Allen B C, Maldonado Y M. Dry Needling Versus Cortisone Injection in the Treatment of Greater Trochanteric Pain Syndrome: A Noninferiority Randomized Clinical Trial[J]. J Orthop Sports Phys Ther,2017,47(4):232-239. |
| 17 | Acosta Pereira A, Magallares Lopez B, Rodriguez De La Serna A. Comparative study between injection with hyaluronic acid and corticosteroids in trochanteritis[J]. 2017,32(4):152-156. |
| 18 | Brinks A, van Rijn R M, Willemsen S P, et al. Corticosteroid injections for greater trochanteric pain syndrome: a randomized controlled trial in primary care[J]. Ann Fam Med,2011,9(3):226-234. |
| 19 | Rompe J D, Segal N A, Cacchio A, et al. Home training, local corticosteroid injection, or radial shock wave therapy for greater trochanter pain syndrome[J]. Am J Sports Med,2009,37(10):1981-1990. |

**Supplemental Figure 1**

The Risk of bias assessment for every included study.


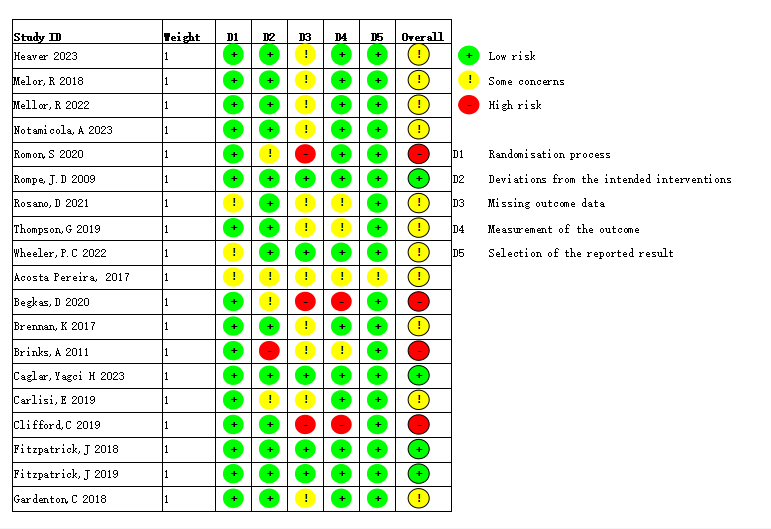


**Supplemental Figure 2**

heterogeneous examination of pain indicators, (A) assessing heterogeneity through subgroup analysis by control group interventions: 1.blank control or sham treatment; 2.physical therapy; 3.injection therapy; (B) conducting heterogeneity assessment for all studies involving VAS pain indicators; (C) conducting heterogeneity assessment for all studies involving NRS pain indicators.

**Supplemental Figure 3**


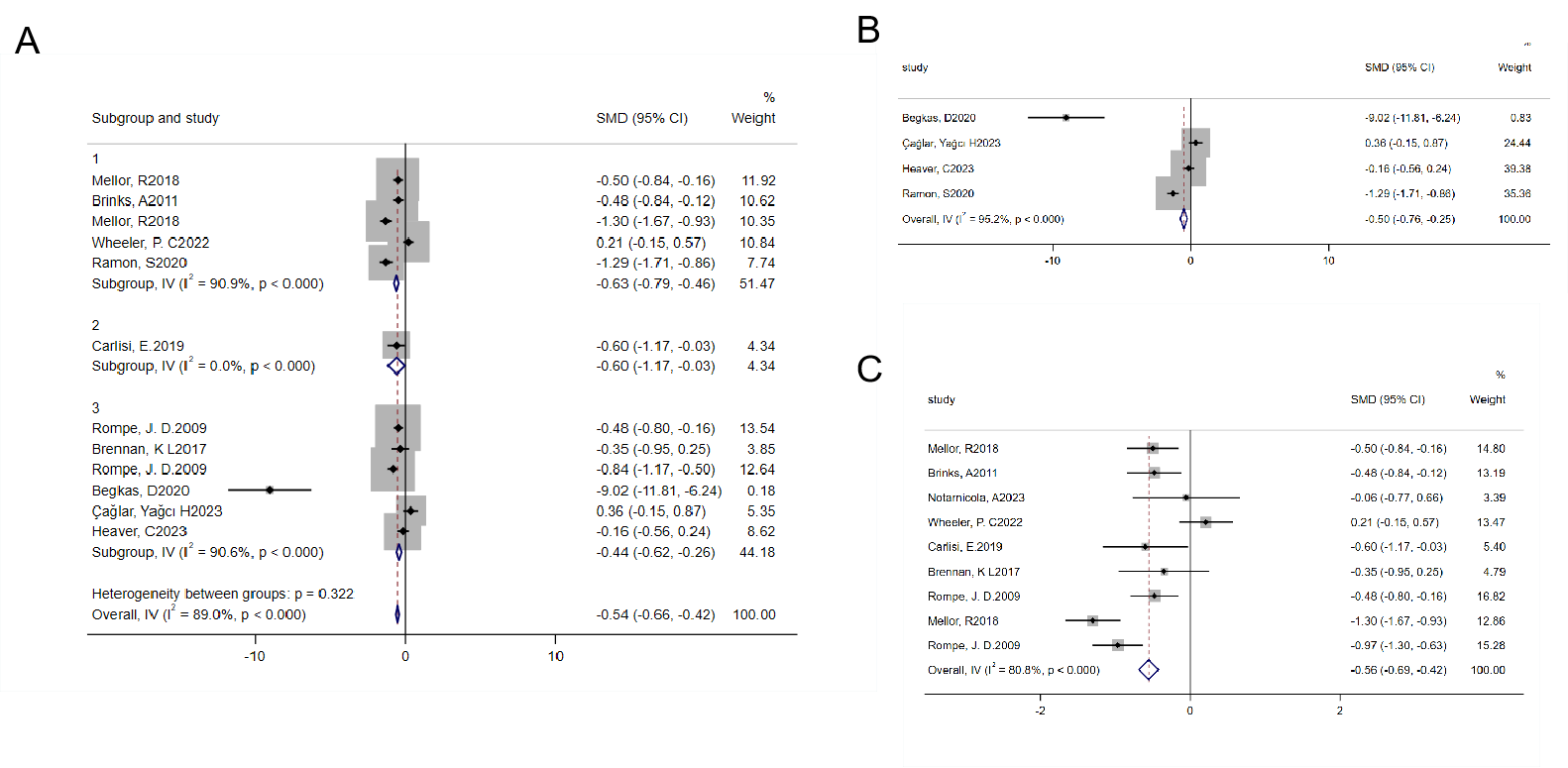
further explored heterogeneity in functional indicators, (A) conducting subgroup analysis by control group interventions: 1) blank control or sham treatment; 2) exercise therapy; 3) injection therapy; (B) assessing heterogeneity across all studies involving HHS functional indicators; (C) evaluating heterogeneity across all studies involving VISA-G functional indicators.

**
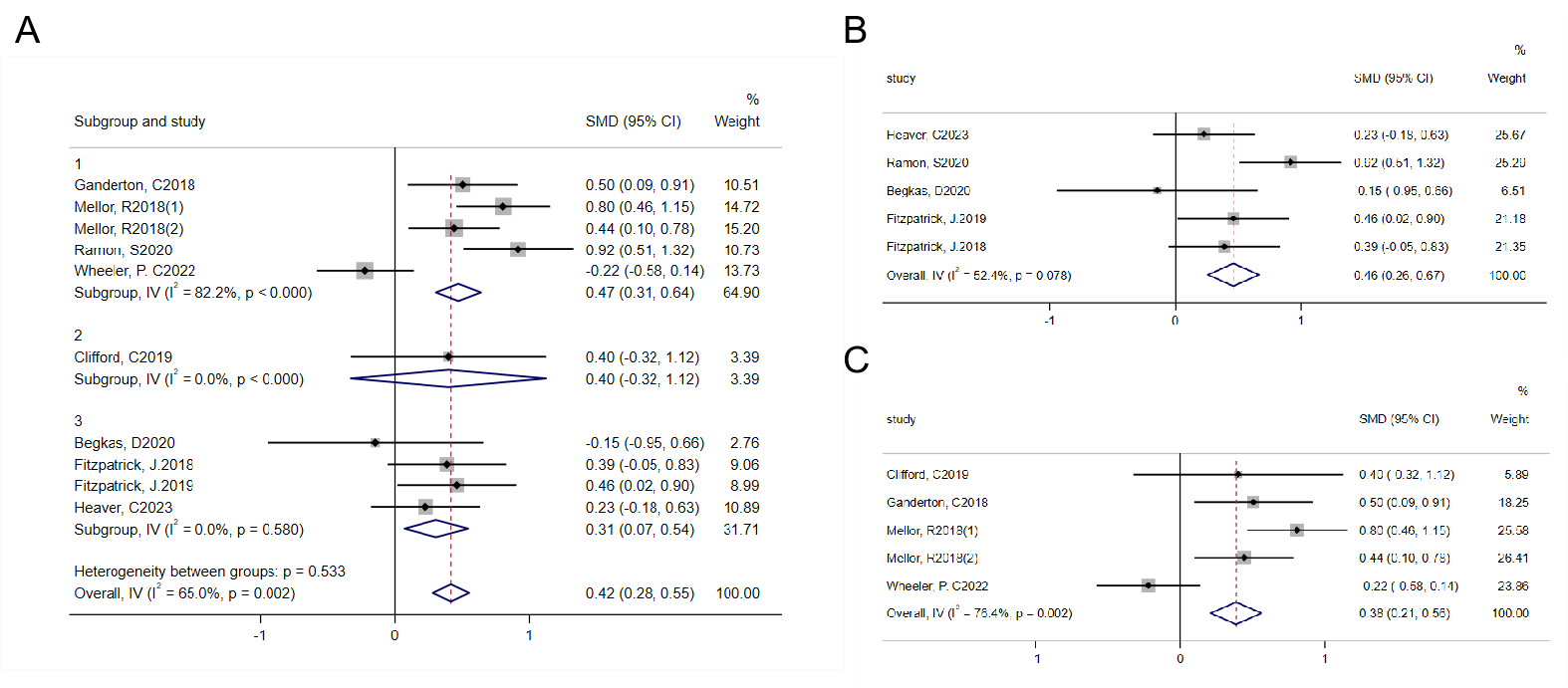
**
